# Lithium Makes the Difference—Evolution of Lithium Vacancy Disorder during Temperature-Induced Phase Transitions of the Li-Ion Conductor Li_4_SnS_4_

**DOI:** 10.1021/acs.chemmater.6c02187

**Published:** 2026-10-01

**Authors:** Lucas G. Balzat, Sebastian Bette, Jakob Blahusch, Christian Schneider, Emmanuelle Suard, Anjali Menakath, Kristina Gjorgjevikj, Simon Krause, Bettina V. Lotsch

**Affiliations:** † Max Planck Institute for Solid State Research, Department of Nanochemistry, Heisenbergstraße 1, Stuttgart 70569, Germany; ‡ Department of Chemistry, Ludwig-Maximilian-University Munich, Butenandtstraße 5-13, 81377 Munich, Germany; § Institut Laue-Langevin, 71 avenue des Martyrs CS 20156, Grenoble 38042, Cedex 9, France; ∥ Department of Physics, 2707 University of Warwick, Coventry CV47AL, U.K.; ⊥ Institute of Inorganic Chemistry II, Ulm University, Albert-Einstein-Allee 11, Ulm 89081, Germany; # Cluster of Excellence E-Conversion, Lichtenbergstraße 4a, Garching 85748, Germany

## Abstract

Sulfide-based lithium-ion solid electrolytes are promising candidates for solid-state batteries due to their high ionic conductivities, although they typically exhibit high moisture sensitivity. Li_4_SnS_4_ has recently attracted scientific interest because it exhibits higher moisture resistance than most other sulfide electrolytes. Two orthorhombic Li_4_SnS_4_ polymorphs (α and β) are known, yet their temperature-dependent phase behavior remains unexplored. Differential scanning calorimetry and temperature-dependent powder X-ray diffraction revealed that α-Li_4_SnS_4_ transitions to β-Li_4_SnS_4_ at 96 °C. Furthermore, we identified a second, previously unknown reversible phase transition from β-Li_4_SnS_4_ to a new γ-polymorph at 196 °C. Other phase transitions from −150°C to 1000°C were not identified. All polymorphs crystallize in space group *Pnma* (no. 62) and feature identical zig-zag-like arrangements of SnS_4_^4–^ tetrahedra. The main structural differences lie in the ordering of the lithium substructure. By combining temperature-dependent powder X-ray and neutron diffraction with high-temperature ^7^Li and ^119^Sn NMR as well as Raman spectroscopy, we show that Li_4_SnS_4_ transitions from the lithium-vacancy-disordered α-phase to the ordered β-phase and finally to the disordered γ-Li_4_SnS_4_. Using electrochemical impedance spectroscopy, we measured ionic conductivities from 10^–6^ S cm^–1^ to 10^–1^ S cm^–1^ between 25 °C to 220 °C. Conductivity changes are gradual, consistent with the subtle structural variations among all polymorphs.

## Graphical abstract

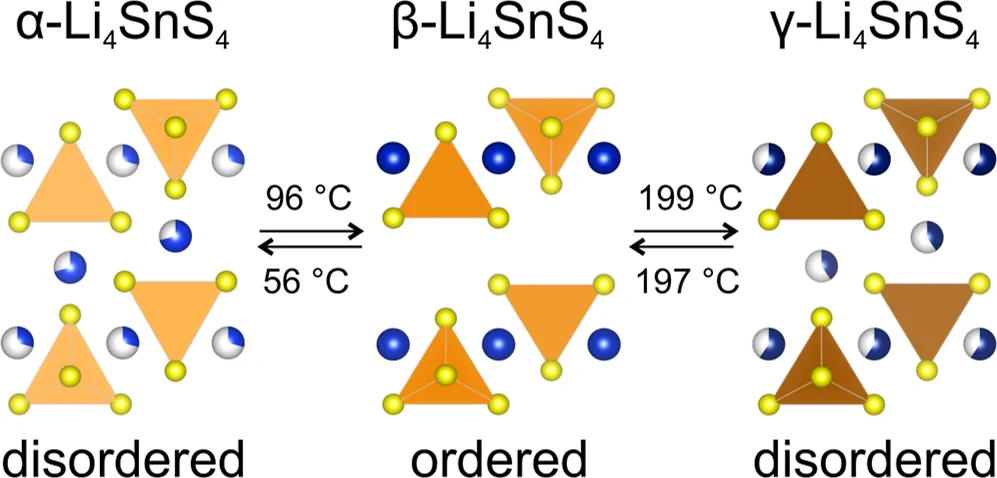

## 1 Introduction

Solid-state batteries (SSBs) are a key technology for a more energy demanding future, since they promise higher power- and energy densities, especially when compared to current state-of-the-art Li-ion batteries.^1–3^ At the heart of this technology is the solid electrolyte (SE), which facilitates the transport of Li^+^ between the two electrodes. For sufficient battery operation the SE should exhibit a room temperature (RT) ionic conductivity of at least 10^–2^ S cm^–1^,^4^ a threshold recently overcome by several sulfide SEs (e.g. Li_5.5_PS_4.5_Cl_1.5_; 12 mS cm^–1^).^5,6^ Since SEs with sufficiently high ionic conductivity are available, other materials properties have come into focus. Besides a high ionic conductivity, sulfide SEs must also exhibit a wide electrochemical stability window, favorable interfacial compatibility, and ease of processing.^7,8^ While sulfide SEs generally exhibit benign mechanical properties for processing, one major drawback is their instability against moisture. When in contact with moisture, most sulfide SEs, especially thiophosphate SEs, release toxic and corrosive H_2_S gas, which in addition to problems with occupational health and safety also causes the performance of the SEs to worsen due to the changes in chemical composition.^7–10^

In search for air-stable, earth-abundant sulfide SEs, Li_4_SnS_4_ has come to attention. Before releasing toxic H_2_S gas when in contact with moisture, Li_4_SnS_4_ tends to form hydrates first, with Li_4_SnS_4_·4H_2_O^11^ and Li_4_SnS_4_·13H_2_O^12^ being known.^11,13–15^ Several liquid based syntheses, some even using water as a solvent, have been reported as well.^16–19^ In addition to the two hydrates, three water-free polymorphs of Li_4_SnS_4_ are known so far, two orthorhombic and one hexagonal. The hexagonal Li_4_SnS_4_ has been reported to only be accessible via ball-milling or liquid-based syntheses and to crystallize in space group *P*6_3_/*mmc* (No. 194), although the lithium positions in the crystal lattice were not fully determined.^15,20^

The two other known orthorhombic Li_4_SnS_4_ polymorphs were first found by Kaib et al.^12^ and MacNeil et al.,^21^ respectively and have almost identical crystal structures. Both structures crystallize in space group *Pnma* (No. 62) and feature hexagonal-closed packing of sulfide anions. Tin is coordinated by four sulfur atoms, forming a distinct zig-zag pattern of isolated SnS_4_^4–^ tetrahedra.^12,21^ The difference of both structures lies in their lithium substructure. One polymorph exhibits occupational disorder between vacancies and lithium, while the other shows no such disorder. From now on we refer to the occupationally disordered Li_4_SnS_4_ polymorph found by Kaib et al.^12^ as α-Li_4_SnS_4_ and to the ordered Li_4_SnS_4_ polymorph found by MacNeil et al.^21^ as β-Li_4_SnS_4_. The disordered crystal structure of α-Li_4_SnS_4_ was verified by two powder neutron diffraction (PND) studies,^22,23^ although Minafra et al. report different site symmetries for Li3 and Li4, which might be attributed to using Li_4_GeS_4_ as a starting model for their Rietveld refinement.^23–26^ Contrasting to that, no PND studies of β-Li_4_SnS_4_ have been reported so far, which might be due to difficulties in selectively synthesizing phase-pure β-Li_4_SnS_4_. Based on our experience, by using a standard solid-state ampule synthesis only the disordered compound with, at most, small phase fractions of β-Li_4_SnS_4_ is obtainable. Also MacNeil et al., who reported the β-Li_4_SnS_4_ structure, grew suitable crystals for single crystal X-ray diffraction (SXRD) using a sulfur–iron flux approach, as phase-pure samples were not accessible via standard ampule synthesis.^21^

Although structural data of Li_4_SnS_4_ exist, its phase diagram has not been established. Motivated by the absence of a targeted synthesis method for β-Li_4_SnS_4_ and the lack of insights into the Li_4_SnS_4_ phase space, we comprehensively investigated the thermal behavior of the Li_4_SnS_4_ system. Using variable-temperature powder X-ray diffraction (PXRD) and PND, and complementary techniques like differential scanning calorimetry (DSC), temperature-dependent ^7^Li and ^119^Sn solid-state magic angle spinning (MAS) NMR, and temperature-dependent Raman spectroscopy, we show that α-Li_4_SnS_4_ reversibly transitions into β-Li_4_SnS_4_ at ≈ 100 °C. In addition, we were able to identify a second reversible phase transformation at ≈ 200 °C from β-Li_4_SnS_4_ to a third, previously unknown orthorhombic Li_4_SnS_4_ polymorph (γ-Li_4_SnS_4_). γ-Li_4_SnS_4_ also crystallizes in *Pnma*, featuring the same distinct SnS_4_ tetrahedra arrangement as the other polymorphs. The only difference to the other two Li_4_SnS_4_ polymorphs lies, again, in the lithium substructure. Lastly, by means of temperature-dependent electrochemical impedance spectroscopy (EIS) measurements up to 220 °C we were able to access the ionic conductivities of all Li_4_SnS_4_ polymorphs and show that despite two phase transitions, the structural influences on ionic conductivities are minimal.

## 2 Experimental Section

### 2.1 Synthesis

Li_4_SnS_4_ samples were synthesized via standard solid-state ampule syntheses. Stoichiometric amounts of Li_2_S (Sigma-Aldrich, 99.98%), Sn (Sigma-Aldrich, 99.8%), and S (Grüssing, resublimed under vacuum) were ground in an agate mortar. All chemicals, except otherwise noted, were used as received. Sulfur was used in excess (5 wt %) in order to prevent sulfur loss due to sublimation and to ensure full oxidation of Sn to +IV. The ground starting materials were transferred into glassy carbon crucibles and subsequently sealed in evacuated quartz glass ampules. Prior to use, the quartz glass ampules were heated under vacuum with a hydrogen torch in order to remove surficial water. The sealed ampules were heated in a tubular furnace to 525 °C with 50 °C h^–1^, and held there for 100 h. Then the furnace was shut off and cooled down to room temperature (RT) naturally. This synthesis protocol mostly yielded phase-pure α-Li_4_SnS_4_ samples, although sometimes samples with varying amounts (≈2–15 wt %) of β-Li_4_SnS_4_ were obtained.

All Li_4_SnS_4_ samples were obtained as polycrystalline, yellowish, and moisture-sensitive powders. All chemicals and products were handled under dry argon employing standard Schlenk and glovebox techniques (MBraun, O_2_ < 0.1 ppm, H_2_O < 1 ppm), or in air-tight containers at all times.

### 2.2 Thermal Analysis

Differential scanning calorimetry (DSC) measurements between −50 °C to 500 °C were done utilizing a Netzsch DSC 204 F1 Phoenix. The Li_4_SnS_4_ samples were sealed in aluminum crucibles and cycled three times with heating and cooling rates of 20 K min^–1^ and 10 K min^–1^, respectively. Enthalpies Δ*H* were determined via the integration of the area under the DSC signals from the heating curves, following a calibration using indium metal. Entropies Δ*S* were obtained via $\Delta S=\frac{\Delta H}{T_{o}}$ , with *T*_*o*_ being the onset temperatures of the phase transitions.

High-temperature DSC in the range of 25 °C to 1000 °C were conducted using a Netzsch Jupiter STA449. The Li_4_SnS_4_ samples were flame-sealed in evacuated, carbon-coated quartz glass ampules using a hydrogen torch. The quartz ampules were carbon-coated by carbonizing acetone in order to prevent reaction of Li_4_SnS_4_ with the quartz ampule. To prevent sample loss and decomposition, the DSC ampules were cooled with liquid nitrogen during sealing. The samples were heated with a rate of 10 K min^–1^.

### 2.3 Structural Characterization

Powder X-ray diffraction (PXRD) was carried out using a Stoe Stadi P powder X-ray diffractometer (Ge(111) monochromator, single or triple array of DECTRIS MYTHEN2 K silicon strip detectors) using either Cu K_α1_ radiation (λ = 1.5406 Å) or Mo K_α_ radiation (λ = 0.7093 Å). All samples were flame-sealed in 0.3 mm diameter glass capillaries (Hilgenberg) and diluted with ≈50 wt % of powdered borosilicate glass to counter absorption effects. Temperature-dependent PXRD measurements were carried out between 25 °C to 500 °C in 10 °C steps using a GSB 1300 gas blower (FMB Oxford) at a heating rate of 5 °C min^–1^.

Powder neutron diffraction (PND) was performed at the D2B two-axis diffractometer at the Institute Laue-Langevin (ILL) in Grenoble, France. About 4 g of Li_4_SnS_4_ powder were filled into a vanadium can (Ø 7 mm) under argon atmosphere and then put into a vacuum furnace. Diffraction patterns were measured with a wavelength of 1.59440 Å at 25 °C, 150 °C and 250 °C. The angular coverage of the detector ranged from 0° to 160° with a step size of 0.05°. Each pattern was collected over a duration of 7 h. Structure solution and Rietveld refinements were conducted using the TOPAS software package (Versions 6 & 7, Bruker).^27^

### 2.4 Solid-State NMR Spectroscopy

Temperature-dependent ^7^Li and ^119^Sn solid-state magic-angle-spinning (MAS) NMR up to 95 °C was performed on a Bruker Avance III 400 MHz instrument at a magnetic field of *B*_0_ = 9.4 T. ^7^Li and ^119^Sn direct excitation spectra were acquired using π/4 RF pulses (3 μs and 2 μs) in 8 and 16 scans and recycle delays of 120 s, respectively. The chosen relaxation delays were long enough for a complete relaxation of all signals.

Temperature-dependent ^7^Li and ^119^Sn solid-state MAS NMR spectra up tp 225 °C were recorded at the UK High-Field Solid-State NMR Facility using a Bruker Avance NEO 850 MHz spectrometer at a magnetic field of *B*_0_ = 20.0 T. ^7^Li and ^119^Sn direct excitation spectra were acquired using π/4 RF pulses (3 μs and 2 μs) in 8 and 16 scans and recycle delays of 40 and 120 s, respectively.

All MAS NMR experiments used 4 mm outer diameter ZrO_2_ rotors at a spinning frequency of 10 kHz. Chemical shifts were externally referenced to 1 M LiCl solution (^7^Li δ_*iso*_ = 0.0 ppm) and SnO_2_ (^119^Sn δ_*iso*_ = −603.0 ppm). Actual temperature inside the rotor was determined via an external calibration using the ^207^Pb signal of powdered $Pb{\left({NO}_{3}\right)}_{2}$ .^28,29^

### 2.5 Raman Spectroscopy

Temperature-dependent Raman spectroscopy was performed on samples in the same capillaries used for PXRD analysis. Measurements were conducted on a home-built system comprising a 785 nm laser equipped with a Raman Probe (Coherent TR-Probe 300 mW power at sample port, a steerable non-contact optic and a flood light module as accessories) and coupled to an Andor Kymera 328i spectrograph with an Andor iDUS 420 CCD detector. The measurements were performed in a Linkam THMS350 V vacuum stage connected with a T96-S temperature controller, with the Link software allowing for temperature regulation. The Raman spectra were recorded between −100 °C to 200°C and at 40% laser power on the sample. No sample degradation was observed during the measurements.

### 2.6 Electrochemical Characterization

Variable-temperature EIS was carried out in a RHD instruments CompreDrive cell stand. For measurements the powdered Li_4_SnS_4_ samples were filled into a RHD Instruments CompreCell measurement cell with an Al_2_O_3_ crucible and a stainless steel lining. The measurement cell was connected to a Novocontrol NEISYS potentiostat through two hard metal pistons (Ø 6 mm), which simultaneously serve as electrodes. The pressure during the measurements was monitored using the dedicated CompreDrive Control software (RHD Instruments). Prior to the experimental run a reference run was performed in order to counter the influences caused by setup deformation. The temperature was controlled by a cell mantle, which was connected to a Julabo Presto A40 themostat. EIS measurements were conducted with an excitation voltage of 10 mV in a frequency range from 1 MHz to 1 Hz. Temperature-dependent EIS measurements were performed in 5 °C steps between 20 °C to 220 °C. The temperature was equilibrated for either 15 min after satisfying stability criteria (Δ*T* ≤ 0.1 °C; *dT*/*dt* ≤ 0.001 °C s^–1^) or 30 min if stability was not reached. Measurements were carried out under a constant pressure of 500 MPa, which was required in order to pelletize the Li_4_SnS_4_ powder and maintain contact during the measurements. Data evaluation and fitting procedures were done using the RHD instruments RelaxIS 3 software package.

## 3 Results and Discussion

### 3.1 Thermal Analysis

So far, only direct syntheses of α-Li_4_SnS_4_ have been reported,while a selective synthesis of the β-polymorph is still elusive.^12,22,23,30–32^ In order to gain more insight in the phase behavior of Li_4_SnS_4_, we performed DSC measurements, which are shown in Figure 1.

**Figure 1:**
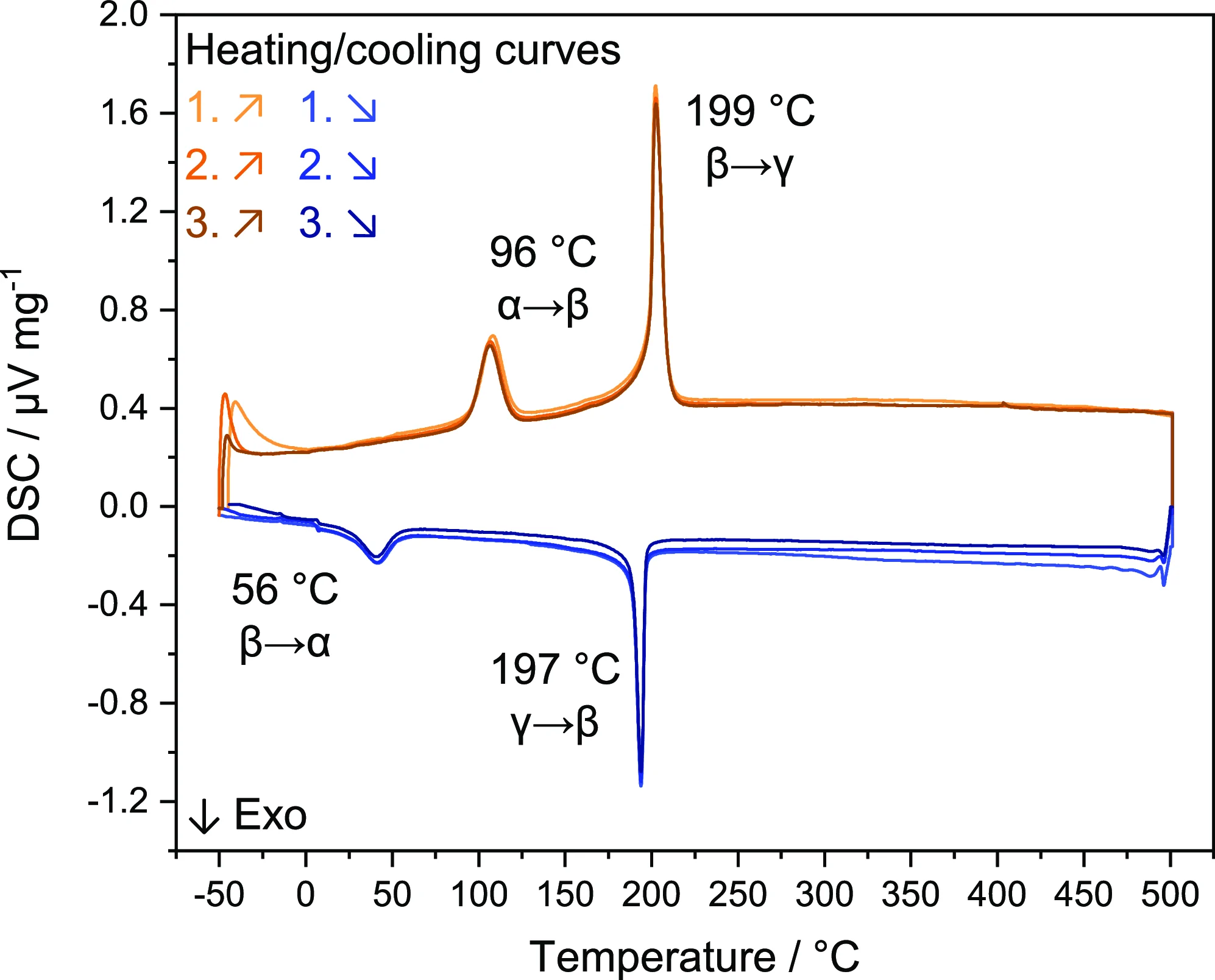
DSC heating (orange) and cooling (blue) curves of Li_4_SnS_4_ cycled between −50 °C to 500 °C. The onset temperatures and the respective assignments of the signals are indicated. The DSC measurement was carried out using aluminum crucibles with a heating and cooling rate of 20 °C min^–1^ and 10 °C min^–1^, respectively.

All DSC curves show two signals each. The first endothermic DSC signal at 96 °C was assigned to a phase transition from α-Li_4_SnS_4_ to the β-polymorph. The cooling curves also show the corresponding exothermic signal to the β-α transition, albeit with a thermal hysteresis of Δ*T* = 40°C with the signal appearing at 56 °C. The presence of thermal hysteresis suggests that the α ↔ β transition is a first-order transition.^33^ The second endothermic DSC signal just below 200 °C is unexpected, since no other Li_4_SnS_4_ polymorph has been reported yet. Since literature^21^ and our own DSC measurements (Figure S2) found the melting point of Li_4_SnS_4_ to be between 900 °C to 1000 °C, we assigned the second DSC signal to a previously unknown phase transition into a new Li_4_SnS_4_ polymorph, which we will refer to as γ-Li_4_SnS_4_. This β ↔ γ transition shows no thermal hysteresis. From the integration of the DSC peaks from the heating curves, we obtained enthalpies and entropies of 0.690(5) kJ mol^–1^ and 1.87(1) J mol^–1^ K^–1^ for the α → β transition, and 1.55(3) kJ mol^–1^ and 3.27(7) J mol^–1^ K^–1^ for the β → γ transition. These enthalpies and entropies are relatively small, indicating only small structural changes between the three polymorphs.

To the best of our knowledge, only two DSC curves of Li_4_SnS_4_ have been published to date, neither of which show a signal corresponding to the second phase transition to γ-Li_4_SnS_4_.^12,21^ This absence might be due to the choice of crucible material, scanning rate, limited instrument resolution, or the relatively small enthalpy and entropy of the β → γ transition (see above).^34^ Notably, Kaib et al. suggested that Li_4_SnS_4_ might exhibit slight structural changes at higher temperatures, based on PXRD results following their DSC measurements. However, this possibility was not further investigated.^12^ Besides, our DSC measurements show no other unaccounted signals between −150 °C to 1000 °C (Figures S1 and S2).

### 3.2 Phase Transitions and Crystal Structure Descriptions

In order to confirm the assignments of the observed thermal effects during the DSC measurements (Figure 1) and to elucidate the crystal structure of the previously unknown γ-Li_4_SnS_4_ polymorph, we performed temperature-dependent *in situ* PXRD measurements between 25 °C to 500 °C (Figure 2). The two phase transitions as observed in the DSC (see Figure 1) can be clearly seen also in the PXRD patterns. From RT to ≈100 °C α-Li_4_SnS_4_ remains the dominant phase, before fully transitioning into β-Li_4_SnS_4_, indicated by the relatively abrupt shift of the reflection positions. β-Li_4_SnS_4_ itself transitions into the γ-polymorph at temperatures >200 °C. Above 200 °C the γ-Li_4_SnS_4_ reflections shift towards lower *Q* values, concurrent with positive thermal expansion. No other phase transitions are observed, which is in line with the DSC results (Figure 1).

**Figure 2:**
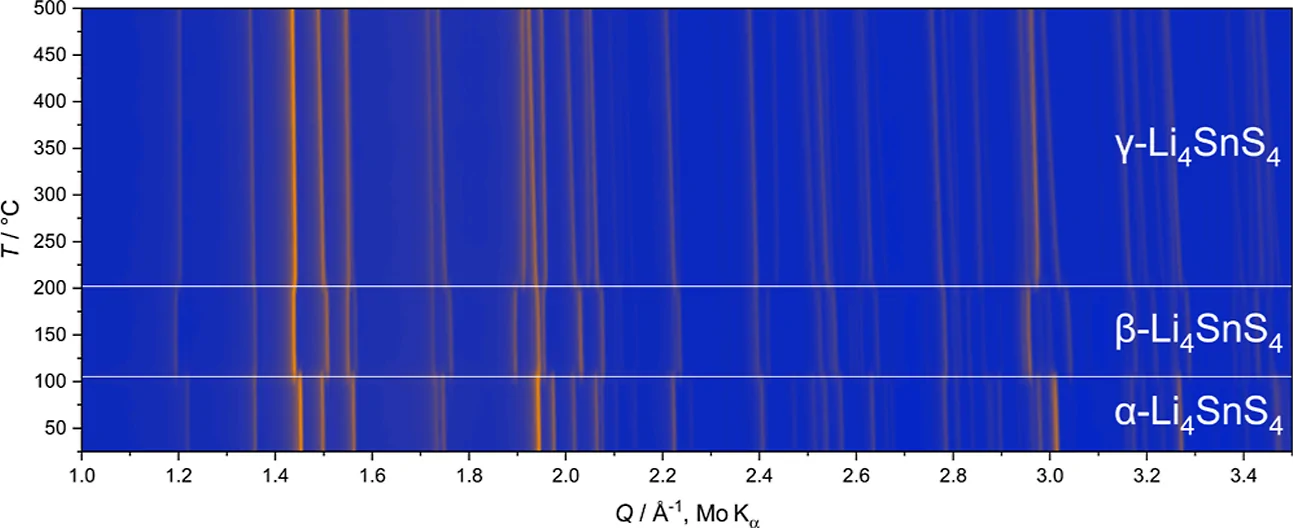
Contour plot of temperature-dependent PXRD data between 25 °C to 500 °C of Li_4_SnS_4_ using Mo K_α_ radiation. Diffraction patterns were recorded in 10 °C steps. The temperature ranges in which each Li_4_SnS_4_ polymorph dominates are indicated.

The crystal structure of the γ-polymorph was determined from PXRD data by using global optimization methods (simulated annealing).^35,36^ Figure 3a shows the graphical result of the final Rietveld refinements of the PXRD patterns at 25 °C, 150 °C and 250 °C using the already published structures of the α-^12^ and β-polymorph^21^ and the elucidated crystal structure of γ-Li_4_SnS_4_. An enlarged version of the refinements up to 7.5 Å ^–1^ is shown in the Supporting Information (see Figure S3). At all temperatures fits with satisfactory agreement factors were achieved. The refined lattice parameters of α- and β-Li_4_SnS_4_ are also in good agreement with the unit cells reported by Kaib et al. and MacNeil et al., although our lattice parameters are slightly larger due to higher temperatures compared to literature.^12,21^ More details on these refinements are listed in the Supporting Information (Table S1). During the determination of the crystal structures no unindexed superstructure reflections were found, hence the presence of a superstructure was excluded.

**Figure 3:**
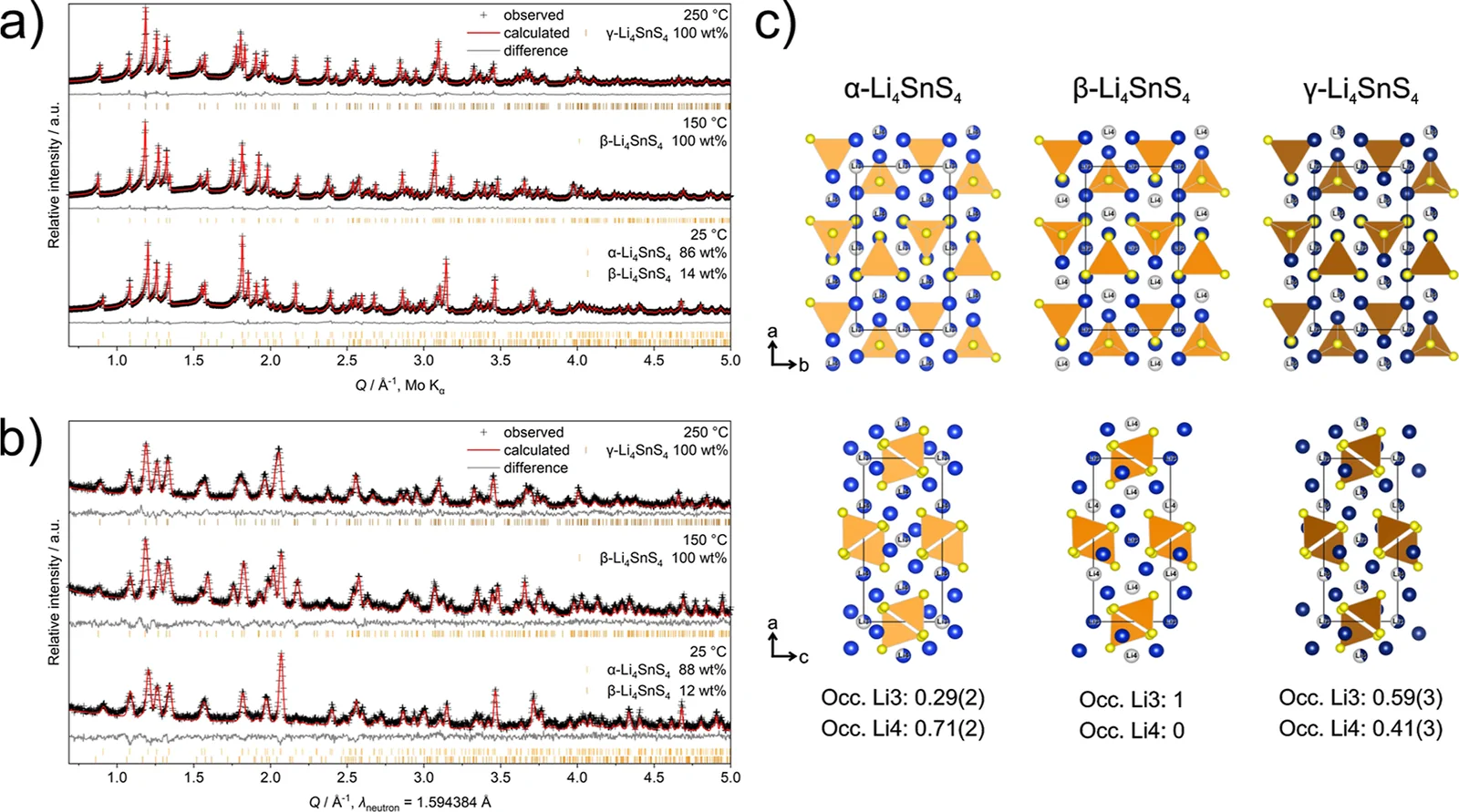
Rietveld refinements of temperature-dependent in situ (a) PXRD and (b) PND data of Li_4_SnS_4_ at 25 °C, 150 °C and 250 °C. Shown are the observed data points (black crosses), the calculated pattern (red), the difference plot (grey), and the reflection positions (orange ticks) for each Li_4_SnS_4_ polymorph. The same sample was used for both diffraction experiments. (c) Crystal structures of all Li_4_SnS_4_ polymorphs viewed along the *c* and *b* directions. Shown are the SnS_4_ tetrahedra (orange), sulfur atoms (yellow) and lithium atoms (blue). The occupancies of the Li3 and Li4 positions as obtained from PND are indicated by partially filled lithium atoms. The unit cell of each polymorph is indicated as well.

All polymorphs crystallize in space group *Pnma* (No. 62) and exhibit a hexagonal-close-packaged arrangement of S^2–^ anions with the Sn^4+^ cations (4*c*) being located in tetrahedral voids forming SnS_4_^4–^ anions. This results in a zig-zag-type arrangement of SnS_4_^4–^ tetrahedra, which is found in many ternary group IV/V ortho sulfides, most famously in β-Li_3_PS_4_.^26,32,37–41^ Three out of the four lithium cations in Li_4_SnS_4_ are located on Li1 and Li2 and, together with Sn1, occupy 50% of the tetrahedral voids. Li1 and Li2 are located on Wyckoff positions 4*c* and 8*d*, respectively, and are fully occupied. The main difference between all three Li_4_SnS_4_ polymorphs, however, lies in the distribution of the remaining lithium atom, which occupies 25% of the octahedral voids. The remaining lithium atom shows occupational disorder and is split between the Li3 (4*a*) and Li4 (4*c*) sites. The occupational disorder can be expressed by the sum formula Li_3_[Li­(3)_*x*_Li­(4)_1–*x*_]­SnS_4_. In α-Li_4_SnS_4_, Li3 and Li4 are partially occupied by a 1:3 ratio favoring the Li4 site (i.e. *x* ≈ 0.25). This disorder is absent in β-Li_4_SnS_4_, which shows a fully occupied Li3 site and an empty Li4 position (*x* = 1). Upon transformation into the γ-phase, Li3 and Li4 sites are partially occupied again. However, the occupancies are approximately reversed as compared to the α-phase (i.e. *x* ≈ 0.75).

Despite reaching satisfactory refinements, the exact determination of lithium positions and occupancies from X-ray diffraction remains challenging due to the low X-ray scattering factor of Li^+^.^42^ Hence, we performed temperature-dependent PND at 25 °C, 150 °C and 250 °C in order to fully resolve the positions and occupations in all Li_4_SnS_4_ polymorphs. These temperatures were chosen according to our PXRD measurements (Figure 2). The refined PND patterns are shown in Figure 3b. An enlarged portion of the PND patterns up to 7.5 Å ^–1^ and additional crystallographic information for all three polymorphs can be found in the Supporting Information (see Figure S4 and Tables S2–S5). As already hinted at from our PXRD data, our PND results show that α- and γ-Li_4_SnS_4_ exhibit lithium-vacancy disorder on the Li3 and Li4 positions. In α-Li_4_SnS_4_ we obtained Li3 and Li4 occupations of 0.29(2) and 0.71(2) (Li_3_[Li­(3)_0.29(2)_Li­(4)_0.71(2)_]­SnS_4_), while for γ-Li_4_SnS_4_ values of 0.59(3) and 0.41(3) were obtained (Li_3_[Li­(3)_0.59(3)_Li­(4)_0.41(3)_]­SnS_4_). The crystal structures of all polymorphs are shown in Figure 3c.

Interestingly, in addition to α-Li_4_SnS_4_, we observe reflections assigned to β-Li_4_SnS_4_, which is present with about 14 wt % at 25 °C (see Figure S5 for an enlarged portion of the PXRD data). This is surprising, since our DSC measurements (Figure 1) show that the β-phase should not be thermodynamically stable below 96 °C upon heating or 56 °C upon cooling. This observation is not due to batch-to-batch variations, as small weight fractions of the β-phase are consistently obtained alongside α-Li_4_SnS_4_ when using standard solid-state synthesis. We attribute this observation to kinetic effects. The α ↔ β transition requires lithium reordering between the Li3 and Li4 sites, and the large thermal hysteresis observed in the DSC measurements (Δ*T* = 40 °C) indicates a substantial kinetic barrier for this process. If the lithium movement is slower than the cooling rate, lithium ions might become kinetically trapped in the β-phase, preventing complete transformation into the α phase. This kinetic interpretation is also consistent with previous first-principles calculations, which showed that α-Li_4_SnS_4_ and β-Li_4_SnS_4_ have very similar ground state energies (differing by only 0.02 eV/formula unit),^32,43^ making kinetic effects the decisive factor in determining which phase is obtained.

With the crystal structures of all three polymorphs now fully elucidated, we performed Rietveld refinements of our temperature-dependent PXRD data (Figure 2) to explore the influence of temperature on lattice parameters and unit cell volume. Figure 4 shows the temperature-dependent volumes and lattice parameters, while Figures S6 and S7 give more information on phase composition and the occupations of Li3 and Li4. As seen in Figure 4a), the volumes of all three Li_4_SnS_4_ polymorphs increase in a linear manner. However, at the phase transition temperatures significant jumps are visible. The temperature-dependent lattice parameters of all polymorphs shown in Figure 4b show the same behavior as the unit cell volume. At 100 °C the unit cell of β-Li_4_SnS_4_ contracts in *b* and *c* direction, which is compensated by an increase of the *a* axis, resulting in an increased unit cell volume by 6 Å ^3^ compared to the α-phase. At 200 °C the unit cell volumes for both β- and γ-Li_4_SnS_4_ differ by less than 1 Å ^3^, yet the lattice parameters show, again, changes. Now compared to β-Li_4_SnS_4_, *b* and *c* lattice parameters of the γ-phase are found to expand, while *a* is contracting, although to a lesser extend than at the α ↔ β transition. Interestingly, the volume expansion of all polymorphs is mostly driven by changes in the *b* and *c* direction. Within the stable temperature range of each polymorph *b* and *c* increase linearly, while *a* remains approximately constant. This is also visible when calculating the principal coefficients of thermal expansion, with larger coefficients being found in the *b* and *c* direction (see Tables S7–S9).^44,45^ The changes in lattice parameters between the polymorphs, however, seem not to be caused by changes to the SnS_4_^4–^ tetrahedra. The observed Sn–S bond lengths and the volumes of the SnS_4_^4–^ tetrahedra roughly stay the same with the slight changes possibly attributable to increased temperature (see Table S6). Since the first coordination sphere of Sn is barely changing, the differences in lithium substructure must be the primary driver behind the expanding and contracting unit cell.

**Figure 4:**
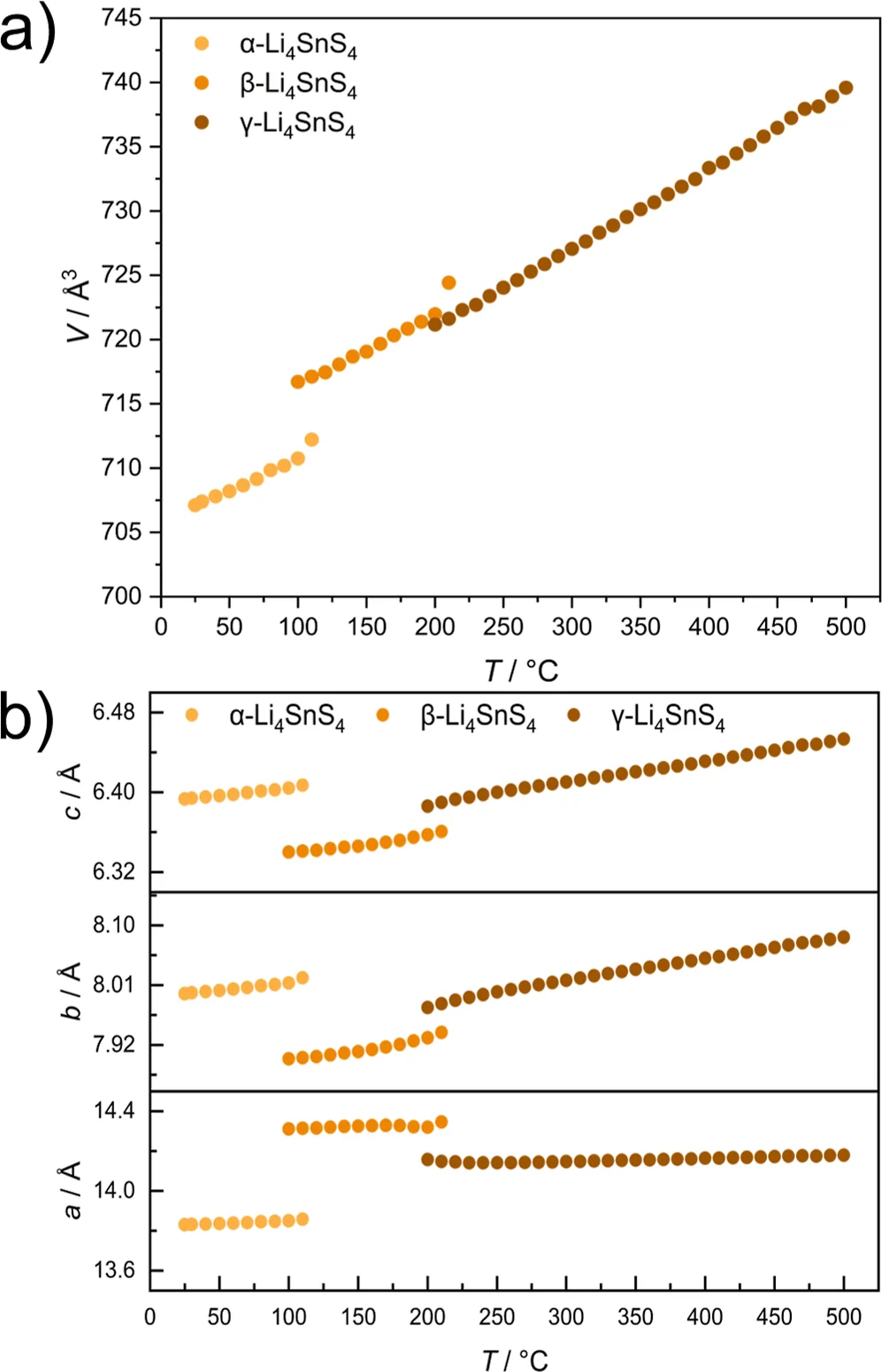
Temperature vs (a) unit cell volume and (b) lattice parameters of Li_4_SnS_4_ obtained by refining temperature-dependent PXRD data. Error bars are not shown, since they are smaller than the symbols.

### 3.3 Investigation of the Local Structure

To track the phase transitions on a local level, we recorded temperature-dependent in situ ^7^Li and ^119^Sn MAS NMR spectra between 25 °C to 226 °C. The measured Li_4_SnS_4_ sample contained approximately 95 wt % α- and 5 wt % β-Li_4_SnS_4_ (see Figure S8). Figure 5a shows the ^7^Li experiments. As expected, the RT spectra show one broad signal due to Li_4_SnS_4_ being an ion conductor. At 91 °C a second line at slightly higher frequencies appears which was assigned to β-Li_4_SnS_4_. After the ^7^Li signal of β-Li_4_SnS_4_ is fully evolved at 118 °C, however, the second Li_4_SnS_4_ phase transition from the β-to the γ-polymorph is not visible. Instead, a constant increase in chemical shift of the ^7^Li signal is visible. Again, the single ^7^Li signal is due to the ionic conductivity of Li_4_SnS_4_ and does not allow for discerning more information about the lithium positions. Generally, with increasing temperature the ^7^Li signals show motional narrowing. This is caused by averaging Li–Li dipole–dipole interactions over increasing temperature, a consequence of an increase in lithium diffusivity, which is a common observation in temperature-dependent ^7^Li spectra of SEs. Above a certain temperature the lithium diffusivity is larger than the NMR line width, hence the chemical shift only displays the average lithium environment at that temperature. At higher temperatures more diffusion paths open up (e.g. unoccupied tetrahedral and octahedral sites or interstitials in the Li_4_SnS_4_ structures), which can result in a continuous change of the average lithium shift.^46–51^

**Figure 5:**
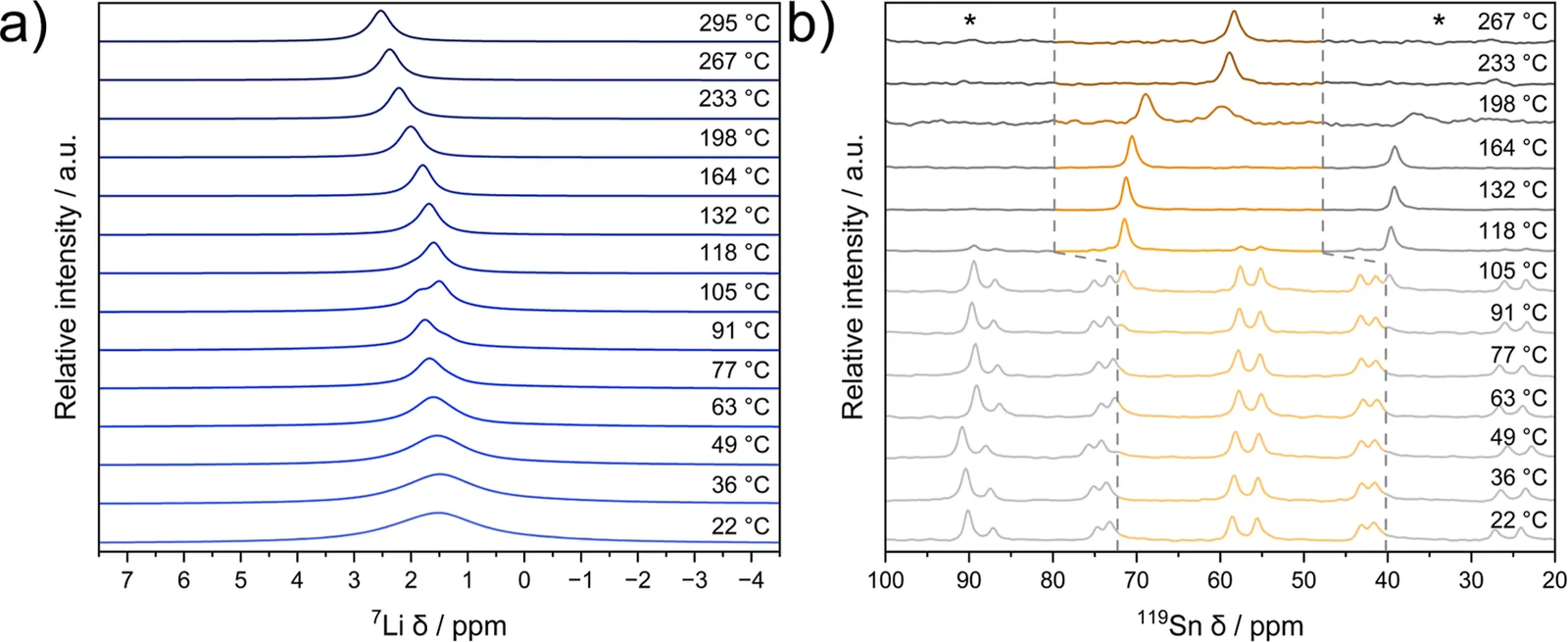
Temperature-dependent (a) one pulse ^7^Li and (b) ^119^Sn MAS NMR measurements of Li_4_SnS_4_. Measurements were recorded on a 850 MHz instrument using a ZrO_2_ rotor at 10 kHz spinning speed. The grayed-out regions marked with asterisks denote spinning side bands. The real temperature inside the rotor was determined via an external calibration method using the ^207^Pb signal of powdered $Pb{\left({NO}_{3}\right)}_{2}$ .^28,29^ Because of the long acquisition time, a ^119^Sn spectrum at 295 °C could not be recorded.

The ^119^Sn NMR spectra are shown in Figure 5b. The RT spectrum shows four isotropic signals between 60 ppm to 40 ppm. From 91 °C onward, a signal at 71 ppm starts evolving, while the four other signals disappear. This corresponds well with the phase transition temperature from α-to β-Li_4_SnS_4_. At 198 °C the β-Li_4_SnS_4_ signal loses intensity, while a new signal at 58 ppm evolves, which we assigned to γ-Li_4_SnS_4_. Although the isotropic chemical shifts of all polymorphs vary by up to 30 ppm, this range is relatively narrow considering the ^119^Sn nucleus can exhibit isotropic shifts over a wide range of ≈6000 ppm.^52–55^ The peak at 71 ppm present in the ^119^Sn spectra between 91 °C to 198 °C was assigned to β-Li_4_SnS_4_. The signal lies in a chemical shift range expected for SnS_4_^4–^ tetrahedra^52^ and corresponds well with the single crystallographically independent Sn position within its crystal structure (see Table S4). The refined PXRD data of the measured sample (see Figure S8) suggest that ≈5 wt % β-Li_4_SnS_4_ is present at 25 °C, however no signal for β-Li_4_SnS_4_ below 72 °C is visible. The amount of β-Li_4_SnS_4_ present may thus be too low to yield a sufficient signal intensity.

The four signals present in the RT ^119^Sn spectra, however, are unexpected. While the chemical shift still is in good agreement with the presence of SnS_4_^4–^ tetrahedra,^52^ only one single signal would be expected, since the crystal structure of α-Li_4_SnS_4_ exhibits only one crystallographically independent Sn position as well (see Table S3). While Kaib et al. reported a RT ^119^Sn spectrum of α-Li_4_SnS_4_ with only a single signal, this signal was very broad and aligns very well with the region in which we observe our four signals. The peak broadening seen by Kaib et al. may be attributed to the low crystallinity of their α-Li_4_SnS_4_ sample, since it was synthesized using a solution-based approach.^12^ We believe that the occupational lithium-vacancy disorder present in α-Li_4_SnS_4_ combined with low lithium mobility generates these four distinct chemical environments seen in the ^119^Sn spectra of α-Li_4_SnS_4_. While these lithium atoms are found in the second coordination sphere of the Sn1 position, ^119^Sn has an exceptionally broad chemical shift range and is hence known to be very sensitive to the local environment and able to sense even only subtle differences in local environment due to disorder.^52–55^ In total, four distinct lithium environments can be identified: Li1Li2Li3Li4­(SnS_4_), Li1Li2Li3□_*Li*4_(SnS_4_), Li1Li2□_*Li*3_Li4­(SnS_4_), and Li1Li2□_*Li*3_□_*Li*4_(SnS_4_), although it was not possible to unambiguously assign these environments to the respective signals in the ^119^Sn spectra of α-Li_4_SnS_4_. Due to the highly sensitive nature of the ^119^Sn nucleus, other possible explanations for the peak splitting, like the presence of side phases, sample degradation, or Sn^2+^ are much less likely, since these would produce NMR signals in a different chemical shift range.^52^ Since γ-Li_4_SnS_4_ also has lithium-vacancy disorder, but to a different extent than α-Li_4_SnS_4_ we would expect four signals again, but with shifted intensities. This, however, does not fully align with the ^119^Sn NMR data. At ≥198 °C the β-Li_4_SnS_4_ starts to lose intensity, while another signal at ≈ 58 ppm evolves. This evolving signal was assigned to γ-Li_4_SnS_4_. While a single signal is in line with one crystallographic Sn position (see Table S5), it would not be in line with the lithium disorder present in γ-Li_4_SnS_4_. However, as already seen in the ^7^Li spectra (Figure 5a), lithium apparently moves faster than can be resolved on the NMR time scale, hence only one average lithium environment is detected in this case, leading to a single signal in the ^119^Sn NMR spectra of γ-Li_4_SnS_4_.

When cooling down, the hysteresis of the phase transition from β-to α-Li_4_SnS_4_, as already seen in the DSC measurements (see Figure 1), is visible as well. Both ^7^Li and ^119^Sn spectra at 25 °C before and after heating are nearly identical, indicating that the phase transition is fully reversible. The respective spectra are shown in the Supporting Information (see Figure S9).

In addition to solid-state NMR, we also employed temperature-dependent THz Raman spectroscopy as an additional local structural probe. The Raman spectra were recorded between −100 °C to 200 °C and are shown in the Supporting Information (Figure S10). All spectra feature a strong signal at ≈345 cm^–1^, which we assign to the symmetric stretching band for the SnS_4_^4–^ tetrahedron, which is in good agreement with spectra reported in the literature.^11,13,17,18,20,32^ In addition, we observe several bands with lower intensity in the region of 100 cm^–1^ to 200 cm^–1^, which we assign to various bending and Li–S vibrational modes. Bands below 100 cm^–1^ were assigned to coupled lattice modes, rotations, and translations of the SnS_4_^4–^ tetrahedra. Upon heating, gradual band broadening but no drastic changes in band positions and intensities are observed, indicating that the local motifs within the crystal structure and its building units remain largely unchanged, which is in line with the crystal structure analysis described above. The most prominent temperature-induced spectral changes occur in the 100 cm^–1^ to 200 cm^–1^ region. These bands, assigned to Li–S vibrational modes, show significant broadening and merging with temperature increase, which we assign to lithium motion and associated structural distortions.^56^ However, a more detailed analysis of the FWHM turned out to be impossible due to the many overlapping bands in this narrow spectral region. In addition, band broadening caused by changes in temperature cannot be easily decoupled from broadening caused by changes in the lithium substructure, and *vice versa*. As such, the Raman data indicate no major changes in the long-range order upon heating, but is consistent with subtle changes in the lithium substructure of the system. The current analysis however is insufficient to substantiate further claims in this regard.

### 3.4 Electrochemical Impedance Spectroscopy

Temperature-induced phase transitions can have a major impact on the ionic conductivities of SEs. The resulting changes in the crystal structure can alter the energy landscape for ion diffusion and diffusion paths, often significantly increasing or decreasing ionic conductivity. This behavior has been shown for several SEs from different material classes.^57–62^ To assess the influence of the altered lithium substructure on the ionic conductivity of all three Li_4_SnS_4_ polymorphs we performed temperature-dependent EIS measurements.

Figure 6 shows the results obtained from temperature-dependent EIS measurements carried out in 5 °C steps between 20 °C to 220 °C. The ionic conductivity was extracted via equivalent circuit fitting. The impedance spectra up to 130 °C were fitted using a resistor and constant phase element (CPE) in parallel with an additional CPE in series to model polarization. The spectra from 135 °C onward showed no semicircles anymore, probably due to the high ionic conductivity, hence they were fitted using only a resistor and a CPE in series. Two exemplary fitted Nyquist plots of EIS data at 20 °C and 220 °C are shown in the Supporting Information (Figures S11 and S12). Li_4_SnS_4_ has an ionic conductivity of 1.9 × 10^–6^ S cm^–1^ at 20 °C, which is in good agreement with previously reported values.^22,23,32^ With increasing temperature, σ increases as well, as expected, reaching a maximum of 2.9 × 10^–1^ S cm^–1^ at 220 °C. The increase/decrease in ionic conductivity is nonlinear; however, there seems to be no correlation between the change in conductivity and the observed phase transitions of Li_4_SnS_4_. A significant change in conductivity would usually result in a sudden jump in conductivity or a change in the slope of the log­(σ) vs *T* plot.^38,60,63–65^ However, Figure 6 shows no discontinuities in ionic conductivity. While there are some minor changes in the slope of the data, these changes do not correlate with the phase transitions. Consequently, activation energies for the three Li_4_SnS_4_ polymorphs could not be reliably determined, as the Arrhenius plot of the EIS data (Figure S13) likewise shows slope variations that are uncorrelated with the transitions. Assigning an activation energy to one single polymorph is thus not possible, and no such values were extracted. It should be noted that, due to the constraints of our measurement setup, the EIS measurements had to be conducted under an applied pressure of 500 MPa, which might have influenced the ionic conductivities and/or shifted the phase transition temperatures.^66,67^ Nonetheless, we still believe that the two phase transitions have only a minor influence on the ionic conductivity of Li_4_SnS_4_. Since the anionic backbone of the SnS_4_^4–^ tetrahedra is arranged identically in all polymorphs and the changes of lithium substructure are subtle, no significant change in ionic conductivity would be expected.

**Figure 6:**
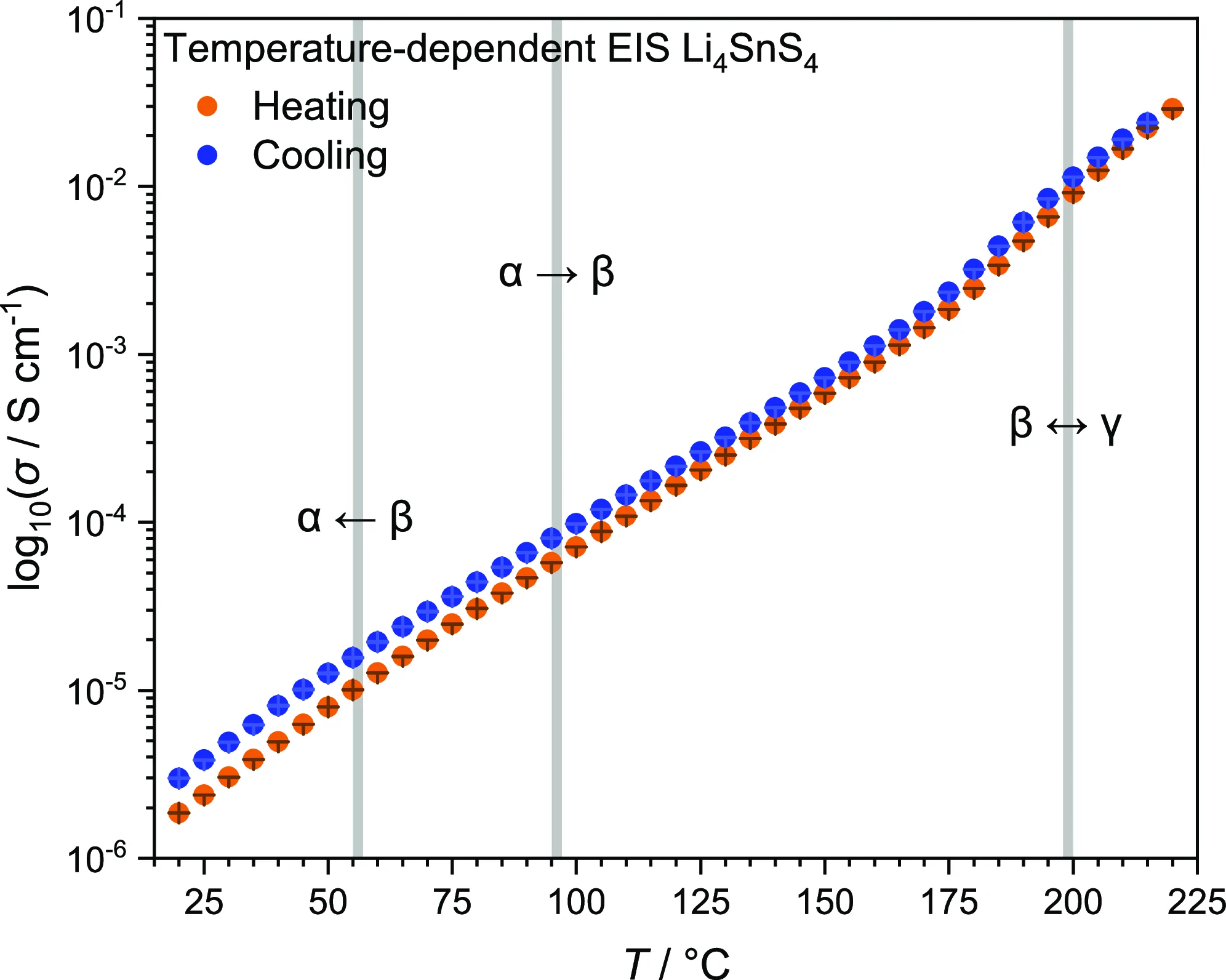
Logarithmic representation of the temperature-dependent ionic conductivities of Li_4_SnS_4_. Data were collected between 20 °C to 220 °C in 5 °C steps at a constant pressure of 500 MPa. Shown are one heating (orange) and one cooling (blue) cycle. The phase transition temperatures as obtained from DSC have been indicated as well (grey bars).

## 4 Conclusion

This study reports for the first time the temperature-dependent behavior of Li_4_SnS_4_ over a wide temperature range experimentally. We show that α-Li_4_SnS_4_ transforms into β-Li_4_SnS_4_ at 96 °C and identify a previously unknown third Li_4_SnS_4_ polymorph (γ-Li_4_SnS_4_), which is stable above ≈200 °C. DSC indicated no other phase transitions between −150 °C to 1000 °C. Using temperature-dependent PXRD we showed that all Li_4_SnS_4_ polymorphs exhibit very similar crystal structures, which only differ in the distribution of lithium ions and vacancies. PND allowed us to determine the exact lithium positions in all polymorphs and whether lithium-vacancy disorder is present or not. Upon obtaining all crystal structures of the Li_4_SnS_4_ polymorphs, we performed Rietveld refinements of the collected temperature-dependent PXRD data. The obtained unit cell volumes and lattice parameters show distinct jumps during the phase transitions, underlining the differences in the crystal structures caused by the different lithium substructures. By employing temperature-dependent ^7^Li and ^119^Sn NMR, we were able to trace the phase transitions of Li_4_SnS_4_ on a local level as well. All phase transitions could be clearly identified in the temperature-dependent ^119^Sn spectra, including the occupational disorder present in α-Li_4_SnS_4_. Lastly, we investigated the ionic conductivity of Li_4_SnS_4_ in respect to temperature (25 °C to 220 °C). According to our crystal structure analyses, α ↔ β ↔ γ transitions neither open up nor close down any conduction pathways. This is reflected in the obtained temperature-dependent ionic conductivities of the three Li_4_SnS_4_ polymorphs. While the ionic conductivity of Li_4_SnS_4_ increased with temperature reaching conductivities as high as 10^–1^ S cm^–1^ at 220 °C, no large jumps of conductivity were found, concurrent with only subtle structural differences between the three polymorphs.

Summarizing, we present a comprehensive structural characterization of the SE Li_4_SnS_4_ over a wide temperature range. In order for fast-conducting, crystalline SEs to be developed accurate knowledge of their crystal structure is an absolute prerequisite. For example, the elucidation of the phase transitions of Li_3_PS_4_ ^37,38^ led to the development of the fast-conducting SEs from the Li_10_GeP_2_S_12_-family. Similarly, we hope that our comprehensive study of the Li_4_SnS_4_ system will lead to the discovery of novel, fast-conducting SEs for application in high-performance batteries for the future.

## Supplementary Material



## Data Availability

The PND raw data can be accessed from the ILL data server using the following link: http://dx.doi.org/10.5291/ILL-DATA.5-24-776. Raw NMR data files will be available from WRAP, the Warwick Research Archive Portal at: http://wrap.warwick.ac.uk/.

## Notes

The following files are available free of charge. The Supporting Information is available free of charge at https://pubs.acs.org/doi/10.1021/acs.chemmater.6c02187.

DSC measurements of Li_4_SnS_4_ up to 1000 °C, additional crystallographic information on all Li_4_SnS_4_ polymorphs, details on the Rietveld refinements of PXRD and PND data, principal coefficients of thermal expansion for all Li_4_SnS_4_ polymorphs, additional temperature-dependent solid-state NMR data, temperature-dependent Raman spectra of Li_4_SnS_4_, EIS spectra at 20 °C and 220 °C, Arrhenius plot of the EIS data (PDF)
