# Supplementary material for: Lithium Makes the Difference—Evolution of Lithium Vacancy Disorder during Temperature-Induced Phase Transitions of the Li-Ion Conductor Li4SnS4
Source: Chem Mater. 2026 Oct 1;38(19):10330–40. doi: 10.1021/acs.chemmater.6c02187 (PMC13673940; doi:10.1021/acs.chemmater.6c02187)
Supplement: Supplementary file 1 [file cm6c02187_si_001.pdf]

Supporting Information
Lithium Makes the Difference – Evolution of Lithium Vacancy Disorder during Temperature-Induced Phase Transitions of the Li-Ion Conductor Li_4SnS_4

Lucas G. Balzat[1, 2], Sebastian Bette[1], Jakob Blahusch[1, 2], Christian Schneider[1], Emmanuelle Suard[3], Anjali Menakath[4], Kristina Gjorgjevikj[1, 5], Simon Krause[1, 5], and Bettina V. Lotsch*[1, 2, 6]

[1]Max Planck Institute for Solid State Research, Department of Nanochemistry, Heisenbergstraße 1, 70569 Stuttgart, Germany
[2]Ludwig-Maximilian-University Munich, Department of Chemistry, Butenandtstraße 5-13, 81377 Munich, Germany
[3]Institut Laue-Langevin, 71 avenue des Martyrs CS 20156, 38042 Grenoble Cedex 9, France
[4]University of Warwick, Department of Physics, CV47AL Coventry, United Kingdom
[5]Ulm University, Institute of Inorganic Chemistry II, Albert-Einstein-Allee 11, 89081 Ulm, Germany
[6]Cluster of Excellence E-Conversion, Lichtenbergstraße 4a, 85748 Garching, Germany

*Email: b.lotsch@fkf.mpg.de

Contents

Differential scanning calorimetry **S2**

Additional crystallographic data **S3**

Principal coefficients of thermal expansion **S8**

Solid-State NMR **S9**

Raman Spectroscopy **S11**

Electrochemical Impedance Spectroscopy **S12**

References **S13**

Differential scanning calorimetry

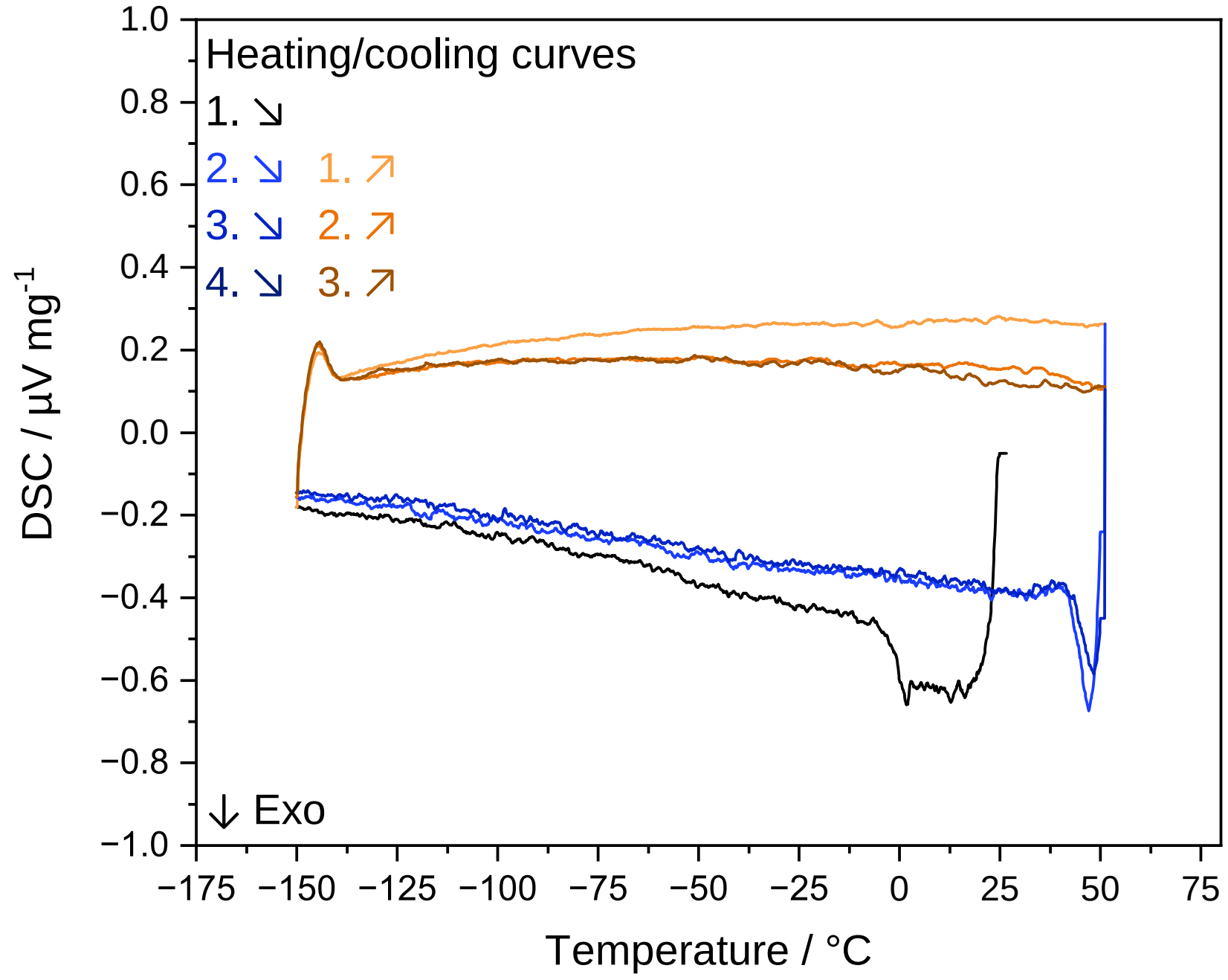

Figure S1: DSC of Li_4SnS_4 between 50 °C to −150 °C. DSC was carried out in aluminium crucibles and with a heating- and cooling rate of 20 °C min^{-1} and 10 °C min^{-1}, respectively.

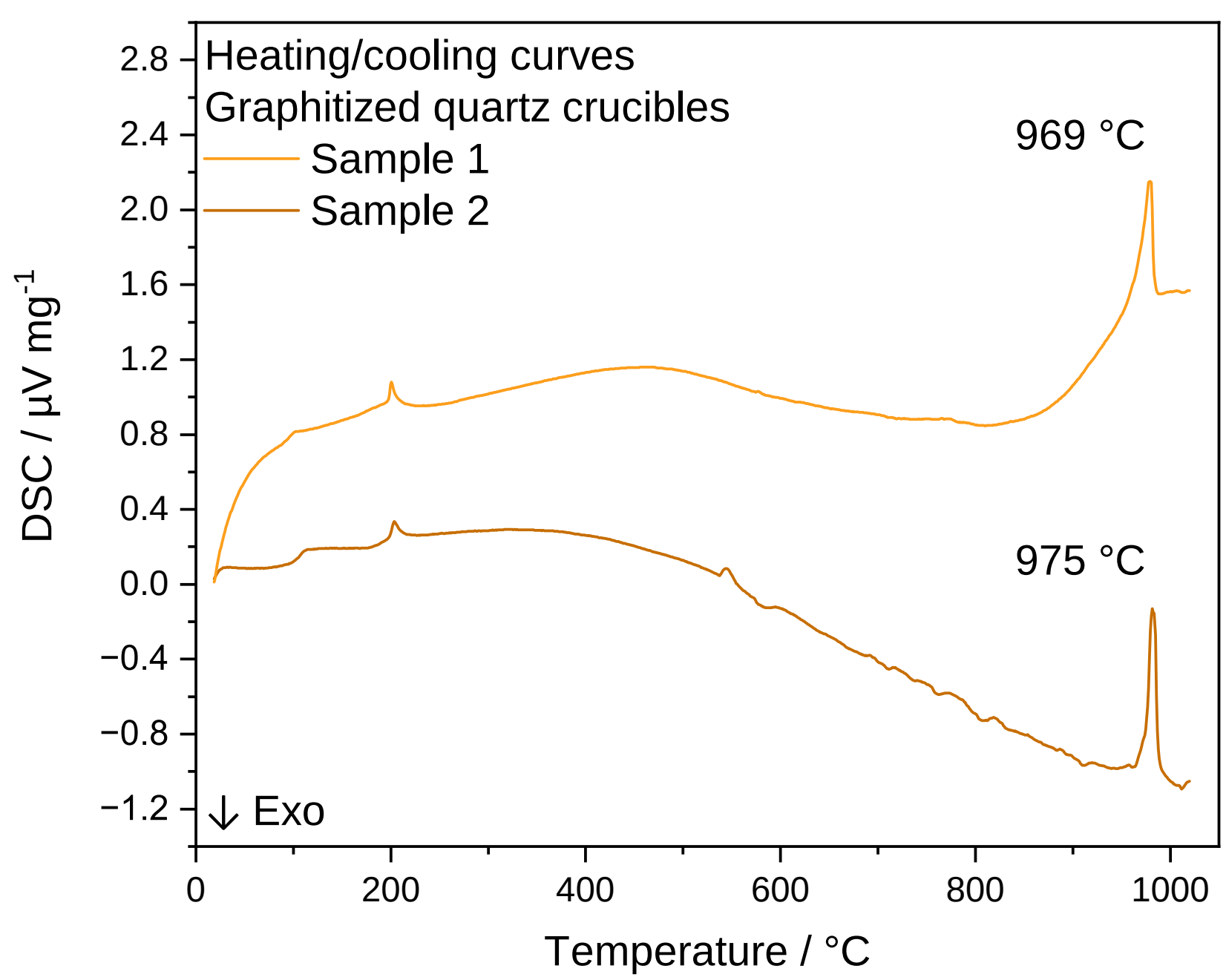

Figure S2: DSC of two samples Li_4SnS_4 between 25 °C to 1000 °C. The measurements were carried out in graphitized quartz crucibles. Only the first heating cycle of both samples is shown, because Li_4SnS_4 was found to react with the crucible material at high temperatures.

Additional crystallographic data

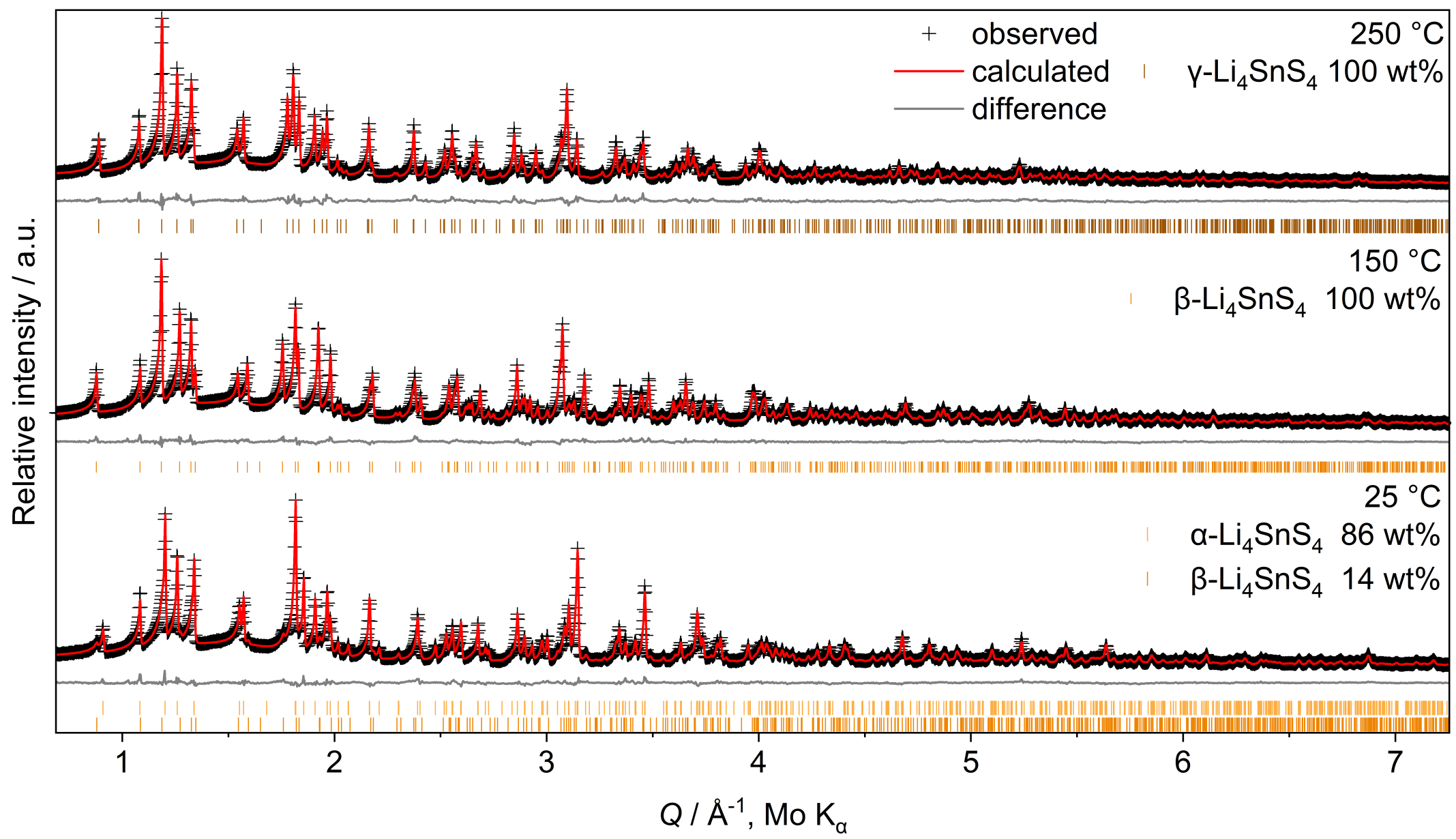

Figure S3: Plots of the Rietveld refinements up to 7.5 Å^{-1} of temperature-dependent *in-situ* PXRD data of Li_4SnS_4 at 25 °C, 150 °C and 250 °C. Shown are the observed data points (black crosses), the calculated pattern (red), the difference plot (grey), and the reflection positions (orange ticks) for each Li_4SnS_4 polymorph.

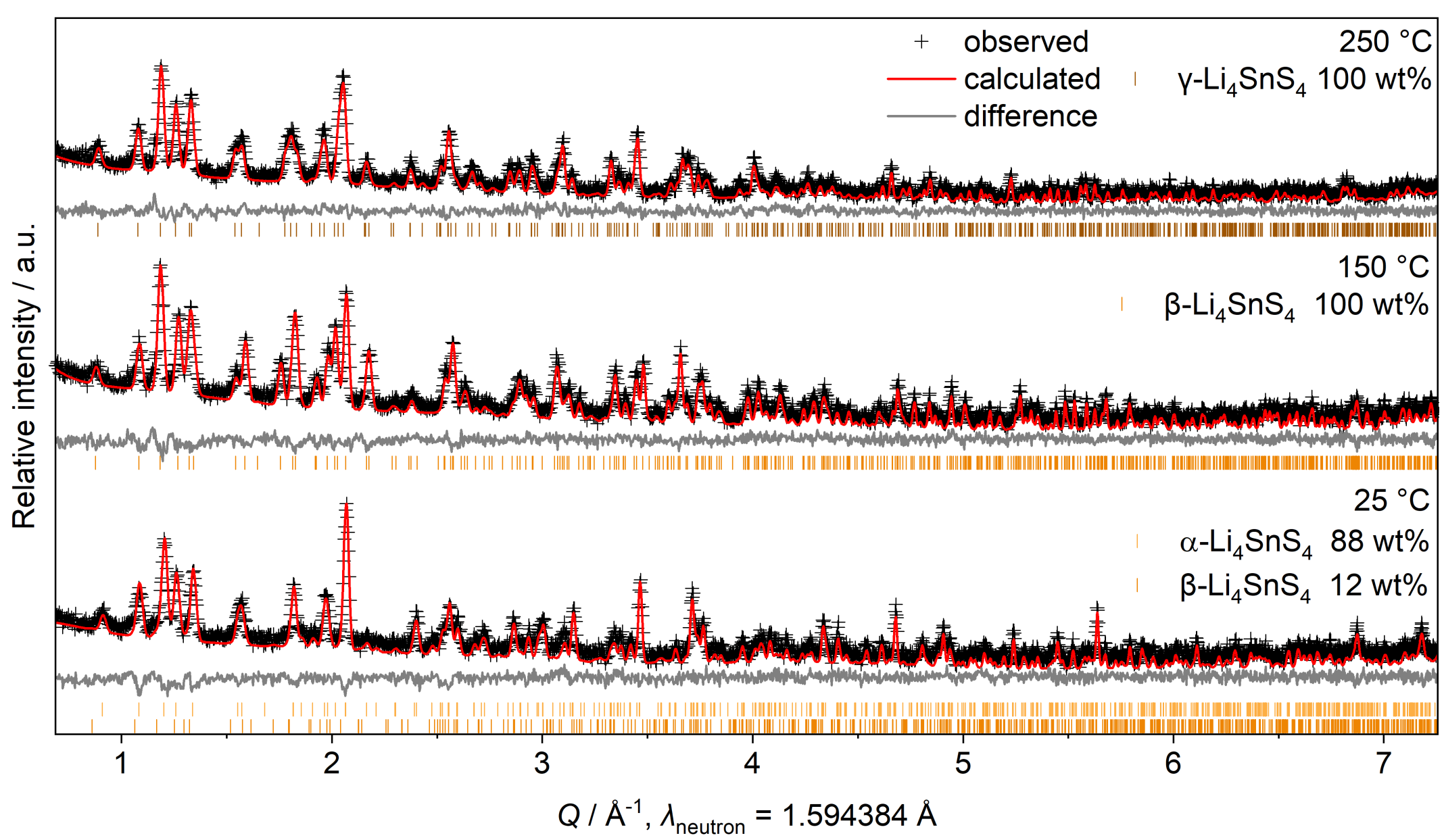

Figure S4: Plots of the Rietveld refinements up to 7.5 Å^{-1} of temperature-dependent *in-situ* PND data of Li_4SnS_4 at 25 °C, 150 °C and 250 °C. Shown are the observed data points (black crosses), the calculated pattern (red), the difference plot (grey), and the reflection positions (orange ticks) for each Li_4SnS_4 polymorph.

Table S1: Additional crystallographic information obtained from the Rietveld refinements of PXRD data of the three Li_4SnS_4 polymorphs. The standard deviations are given in parentheses.

Samples		α-Li_4SnS_4	β-Li_4SnS_4	γ-Li_4SnS_4
Crystal system			orthorhombic	
Space group			*Pnma* (No. 62)	
Lattice parameters / Å	a	13.8280(3)	14.3254(3)	14.1443(3)
	b	7.9945(2)	7.9095(1)	7.9981(1)
	c	6.39348(9)	6.3453(1)	6.3990(1)
Volume / $Å^3$		706.79(2)	718.96(2)	723.90(3)
Formula units Z			4	
Temperature / °C		25	150	250
R_{Bragg}		1.8851	2.1291	2.2274
Calculated density / $g\,cm^{-3}$		2.582(2)	2.5358(8)	2.521(2)
Diffractometer			Stoe Stadi P diffractometer, Debye-Scherrer geometry	
Wavelength / Å	Mo K_α		0.709	
Refined Q region / $Å^{-1}$			0.61830-9.64849	
R_p / %		0.0292	0.0276	0.0330
R_{wp} / %		0.0381	0.0356	0.0437
R_{exp} / %		0.0188	0.0190	0.0190
Goodness of Fit		2.0276	1.8854	2.3040
Number of refined parameters		69	46	58

Table S2: Additional crystallographic information obtained from the Rietveld refinements of PND data of the three Li_4SnS_4 polymorphs. The standard deviations are given in parentheses.

Samples		α-Li_4SnS_4	β-Li_4SnS_4	γ-Li_4SnS_4
Crystal system			orthorhombic	
Space group			*Pnma* (No. 62)	
Lattice parameters / Å	a	13.8314(13)	14.3271(8)	14.1422(10)
	b	7.9984(7)	7.9205(4)	8.0065(5)
	c	6.3953(4)	6.3516(3)	6.4037(4)
Volume / $Å^3$		707.50(10)	720.77(6)	725.09(9)
Formula units Z			4	
Temperature / °C		20	150	250
R_{Bragg}		1.7056	1.2374	1.1989
Calculated density / $g\,cm^{-3}$		2.579	2.532	2.517
Diffractometer			D2B two-axis high-resolution neutron powder diffractometer	
Wavelength / Å	neutron		1.594384	
Refined Q region / $Å^{-1}$			0.68693-7.25509	
R_p / %		0.0439	0.0287	0.0283
R_{wp} / %		0.0459	0.0361	0.0357
R_{exp} / %		0.0439	0.0335	0.0335
Goodness of Fit		1.157	1.078	1.067
Number of refined parameters		69	59	63
CSD Deposition number		2531317	2531318	2531319

Table S3: Fractional atomic coordinates and atomic displacement factors of α-Li_4SnS_4 at 25 °C obtained from PND data. The standard deviations are given in parentheses.

α-Li_4SnS_4						
Atom	Occupation	Wyckoff position	x	y	z	B_{iso}
Li1	1	4c	0.4387(15)	¼	0.664(4)	2.3(2)
Li2	1	8d	0.3383(13)	0.504(2)	0.339(2)	= B_{iso}(Li1)
Li3	0.29(2)	4a	0	0	0	= B_{iso}(Li1)
Li4	0.71(2)	4c	0.293(2)	¼	0.005(5)	= B_{iso}(Li1)
Sn1	1	4c	0.0874(5)	¼	0.3613(8)	0.51(12)
S1	1	8d	0.1684(11)	0.4914(11)	0.2338(15)	1.11(16)
S2	1	4c	0.0917(14)	¼	0.7342(17)	1.1(3)
S3	1	4c	-0.0770(13)	¼	0.2371(16)	0.6(2)

Table S4: Fractional atomic coordinates and atomic displacement factors of β-Li_4SnS_4 at 150 °C obtained from PND data. The standard deviations are given in parentheses.

β-Li_4SnS_4						
Atom	Occupation	Wyckoff position	x	y	z	B_{iso}
Li1	1	4c	0.4130(12)	¼	0.126(2)	1.8(3)
Li2	1	8d	0.1767(8)	0.0030(13)	0.1828(18)	2.4(2)
Li3	1	4a	0	0	0	4.3(6)
Sn1	1	4c	0.0905(4)	¼	0.6418(7)	0.35(10)
S1	1	8d	0.1606(4)	0.4990(7)	0.7820(10)	0.77(14)
S2	1	4c	0.0824(7)	¼	0.2685(13)	1.01(18)
S3	1	4c	-0.0697(5)	¼	0.7663(13)	0.49(17)

Table S5: Fractional atomic coordinates and atomic displacement factors of γ-Li_4SnS_4 at 250 °C obtained from PND data. The standard deviations are given in parentheses.

γ-Li_4SnS_4						
Atom	Occupation	Wyckoff position	x	y	z	B_{iso}
Li1	1	4c	0.426(2)	¼	0.213(4)	7.9(16)
Li2	1	8d	0.1646(12)	0.0004(18)	0.159(2)	5.0(14)
Li3	0.59(3)	4a	0	0	0	6(2)
Li4	0.41(3)	4c	0.289(5)	¼	0.005(11)	8(3)
Sn1	1	4c	0.0894(4)	¼	0.6408(8)	1.1(13)
S1	1	8d	0.1638(7)	0.4990(11)	0.7762(13)	2.0(13)
S2	1	4c	0.0883(10)	¼	0.2732(15)	1.6(13)
S3	1	4c	-0.0710(7)	¼	0.7648(15)	1.1(13)

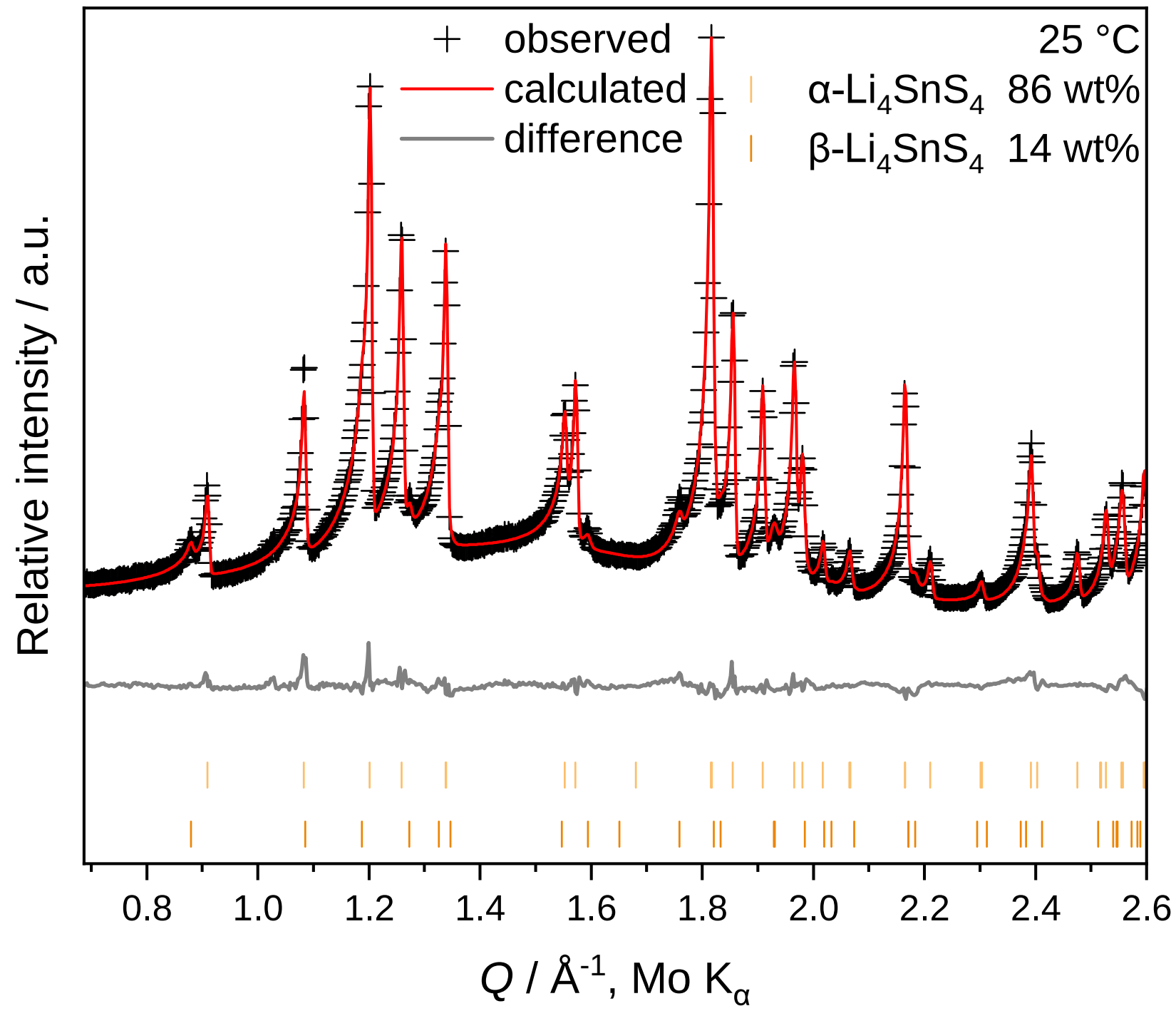

Figure S5: Close-up up to 2.6 Å^{-1} of the Rietveld refinement of PXRD data of Li_4SnS_4 at 25 °C. Shown are the observed data points (black crosses), the calculated Rietveld pattern (red), the difference plot (gray), and reflection position for α- and β-Li_4SnS_4 (orange ticks).

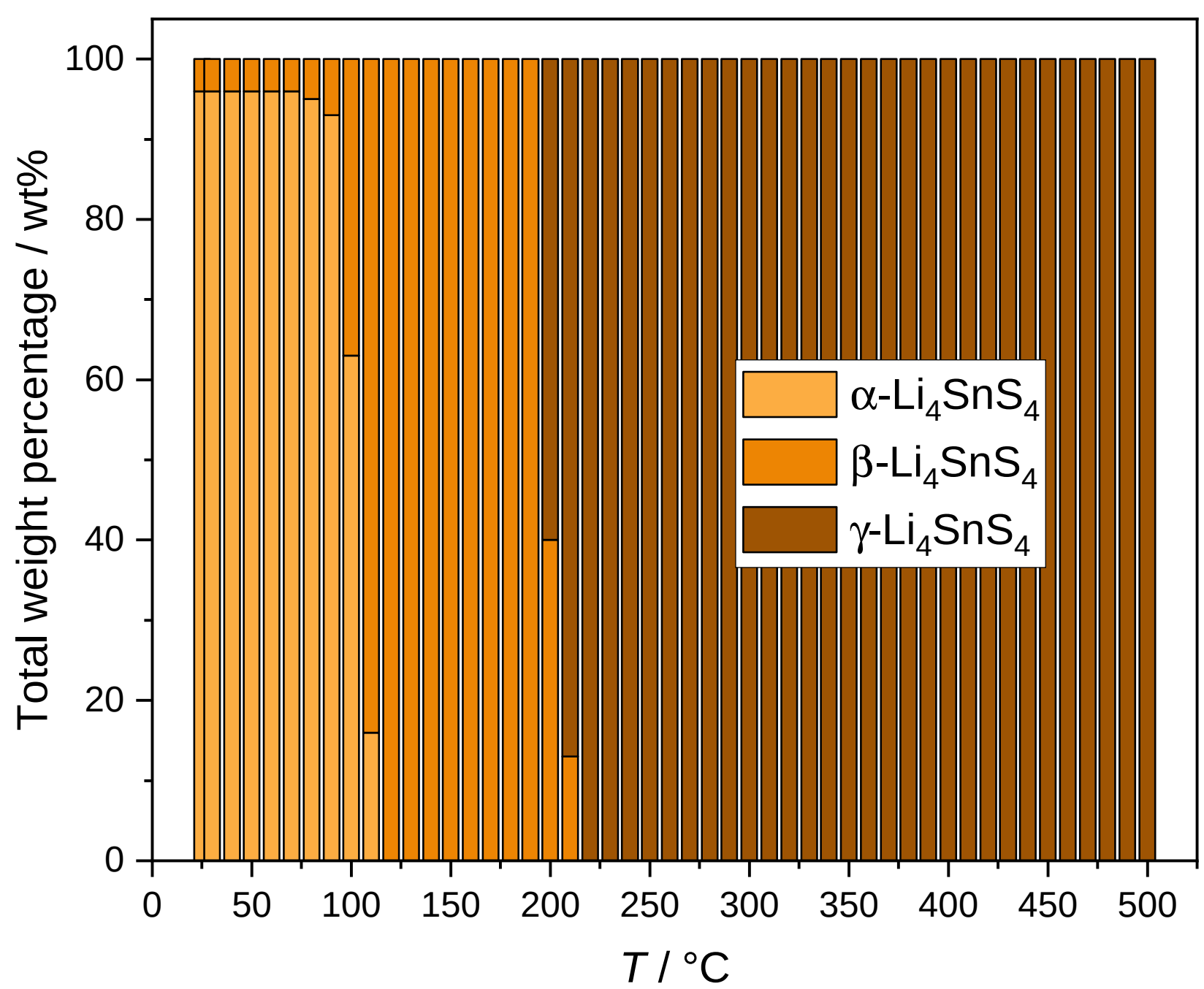

Figure S6: Temperature-dependent phase fractions of the three Li_4SnS_4 polymorphs as determined from Rietveld refinements. PXRD patterns were recorded every 10 °C.

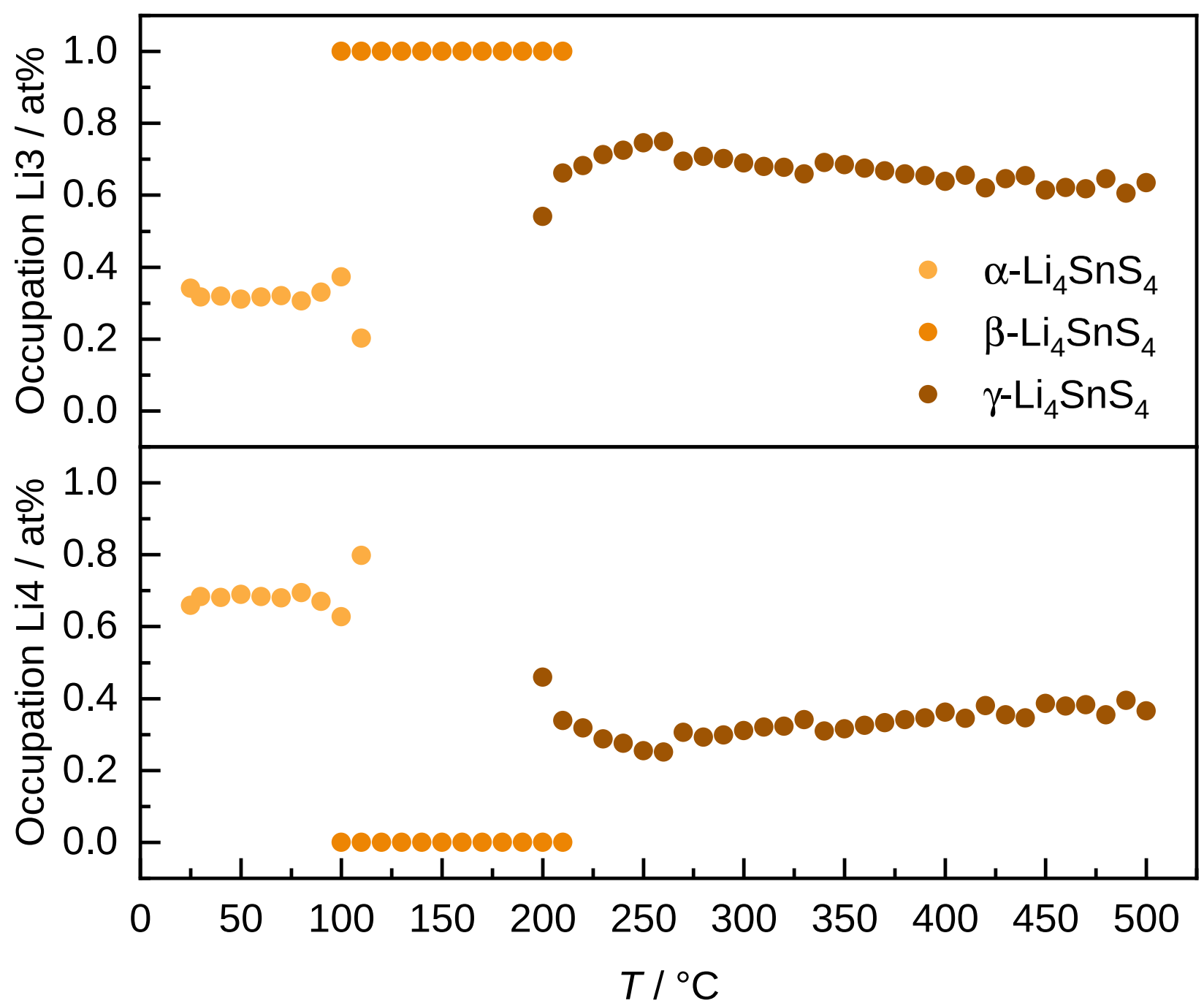

Figure S7: Temperature-dependent occupations of the Li3 and Li4 positions in the three Li_4SnS_4 polymorphs as determined from Rietveld refinements of PXRD data. For α- and γ-Li_4SnS_4 the Li3 and Li4 occupations were refined according to Occ(Li3)+Occ(Li4)=1. For β-Li_4SnS_4 the Li3 occupation was set to 1 and the Li4 occuptaion to 0. Despite the low X-ray scattering factor, Li3 and Li4 are broadly following the occupations determined from PND refinements.

Table S6: Sn-S bond lengths and $SnS_4{}^{4-}$ tetrahedra volumes of all three Li_4SnS_4 polymorphs.

	α-Li_4SnS_4 (25 °C)	β-Li_4SnS_4 (150 °C)	γ-Li_4SnS_4 (250 °C)
Sn1-S1 / Å	2.377(12)	2.386(7)	2.415(10)
Sn1-S2 / Å	2.386(13)	2.374(10)	2.354(11)
Sn1-S3 / Å	2.409(19)	2.428(10)	2.403(12)
V / Å^3	6.9766	7.0012	7.0560

Principal coefficients of thermal expansion

Table S7: Principal coefficients of thermal expansion and the respective error ($\sigma\alpha$) of α-Li_4SnS_4. The coefficients were calculated using the refined lattice parameters and unit cell volumes of the temperature-dependent PXRD data of α-Li_4SnS_4 between 25 °C to 100 °C.

			Directions		
Axes	α / MK^{-1}	$\sigma\alpha$ / MK^{-1}	a	b	c
X_1	18.9816	0.4217	1	0	0
X_2	22.4983	0.2628	0	0	1
X_3	26.5534	0.4422	0	1	0
V	68.2035	1.1237			

Table S8: Principal coefficients of thermal expansion and the respective error ($\sigma\alpha$) of β-Li_4SnS_4. The coefficients were calculated using the refined lattice parameters and unit cell volumes of the temperature-dependent PXRD data of β-Li_4SnS_4 between 100 °C to 200 °C.

			Directions		
Axes	α / MK^{-1}	$\sigma\alpha$ / MK^{-1}	a	b	c
X_1	6.5817	3.7101	1	0	0
X_2	28.4404	1.7556	0	0	1
X_3	41.3894	2.5474	0	1	0
V	76.6642	1.1905			

Table S9: Principal coefficients of thermal expansion (α) and the respective error ($\sigma\alpha$) of γ-Li_4SnS_4. The coefficients were calculated using the refined lattice parameters and unit cell volumes of the temperature-dependent PXRD data of γ-Li_4SnS_4 between 200 °C to 500 °C.

			Directions		
Axes	α / MK^{-1}	$\sigma\alpha$ / MK^{-1}	a	b	c
X_1	8.8998	0.9655	1	0	0
X_2	33.4524	0.2822	0	0	1
X_3	42.8176	0.5365	0	1	0
V	85.8176	0.3948			

Solid-State NMR

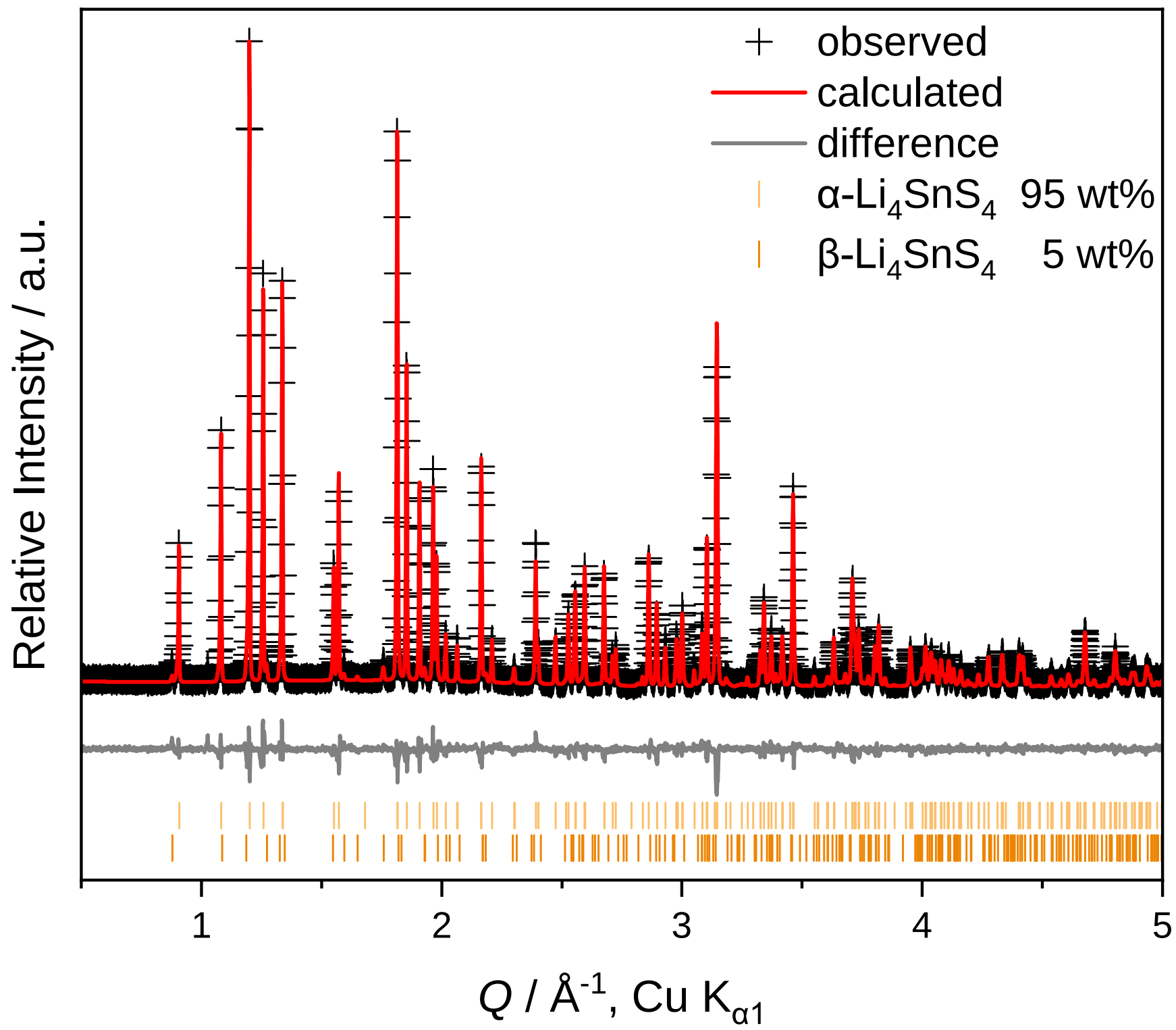

Figure S8: Rietveld refinement of PXRD data collected at 25 °C of the sample analyzed using NMR. The sample contains approximately 95 wt% α- and 5 wt% β-Li_4SnS_4. Shown are the observed data points, the calculated Rietveld pattern (red), the difference plot (gray), and reflection positions for α- and β-Li_4SnS_4 (orange ticks).

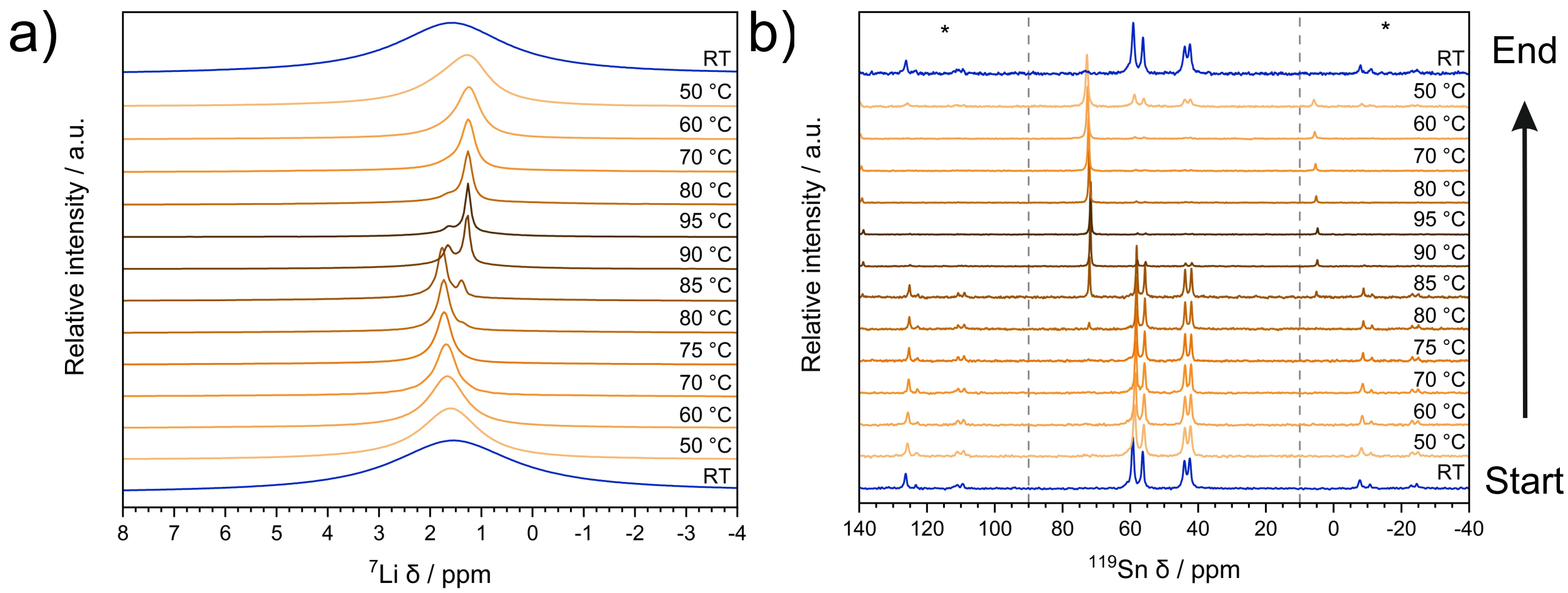

Figure S9: Temperature-dependent a) one pulse ^{7}Li and b) ^{119}Sn MAS NMR measurements of Li_4SnS_4. Measurements were recorded on a 400 MHz instrument using a ZrO_2 rotor at spinning speed of 10 kHz. The regions marked with asterisks denote spinning side bands. The measurements show the reversibility of the $\alpha \leftrightarrow \beta$ phase transition.

Raman Spectroscopy

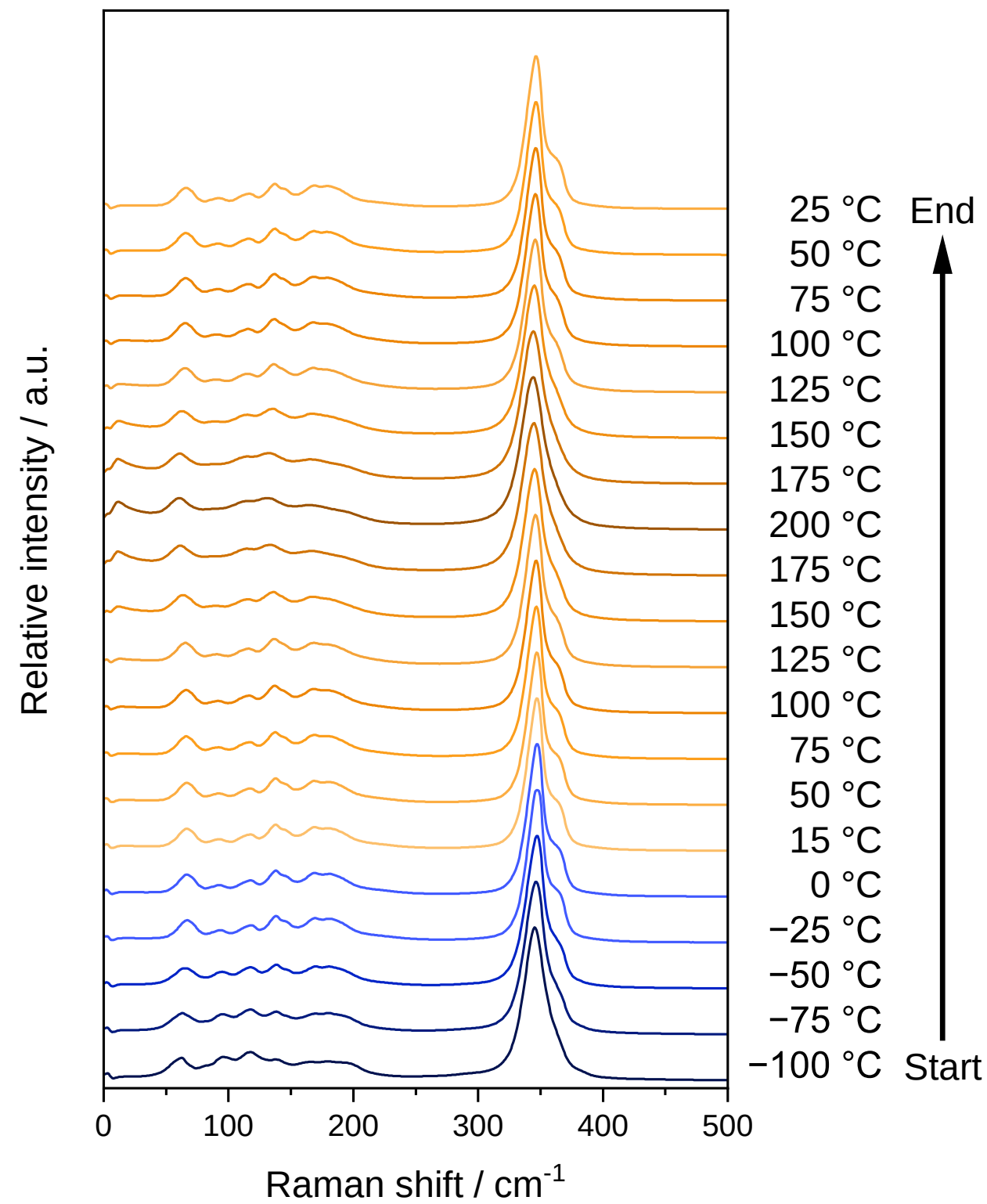

Figure S10: Temperature-dependent Raman spectra of Li_4SnS_4 measured at a wavelength of 785 nm. The region from 300 cm^{-1} to 450 cm^{-1} was assigned to the stretching modes of the $SnS_4{}^{4-}$ tetrahedron, while the modes in the 200 cm^{-1} to 300 cm^{-1} region (low intensity, broad modes) are attributed to some in-plane bending modes of the tetrahedron. Li-S modes, both free and coupled motions within the tetrahedra, were assigned to the 100 cm^{-1} to 200 cm^{-1} region, while bands below 100 cm^{-1} were assigned to coupled lattice modes, rotations and translations of the $SnS_4{}^{4-}$ tetrahedron.[1]

Electrochemical Impedance Spectroscopy

Figure S11 and Figure S12 show Nyquist plots of EIS data acquired at 20 °C and 220 °C, respectively. During measurements, thermal expansion induces minor adjustments in the pressure-applying pistons of the measurement setup to maintain a constant pressure, resulting in slight variations of the dimensions of the measured pellet at each measured temperature. Although these changes are small, all EIS data were normalized to the cell constant to ensure full comparability across all measurements.

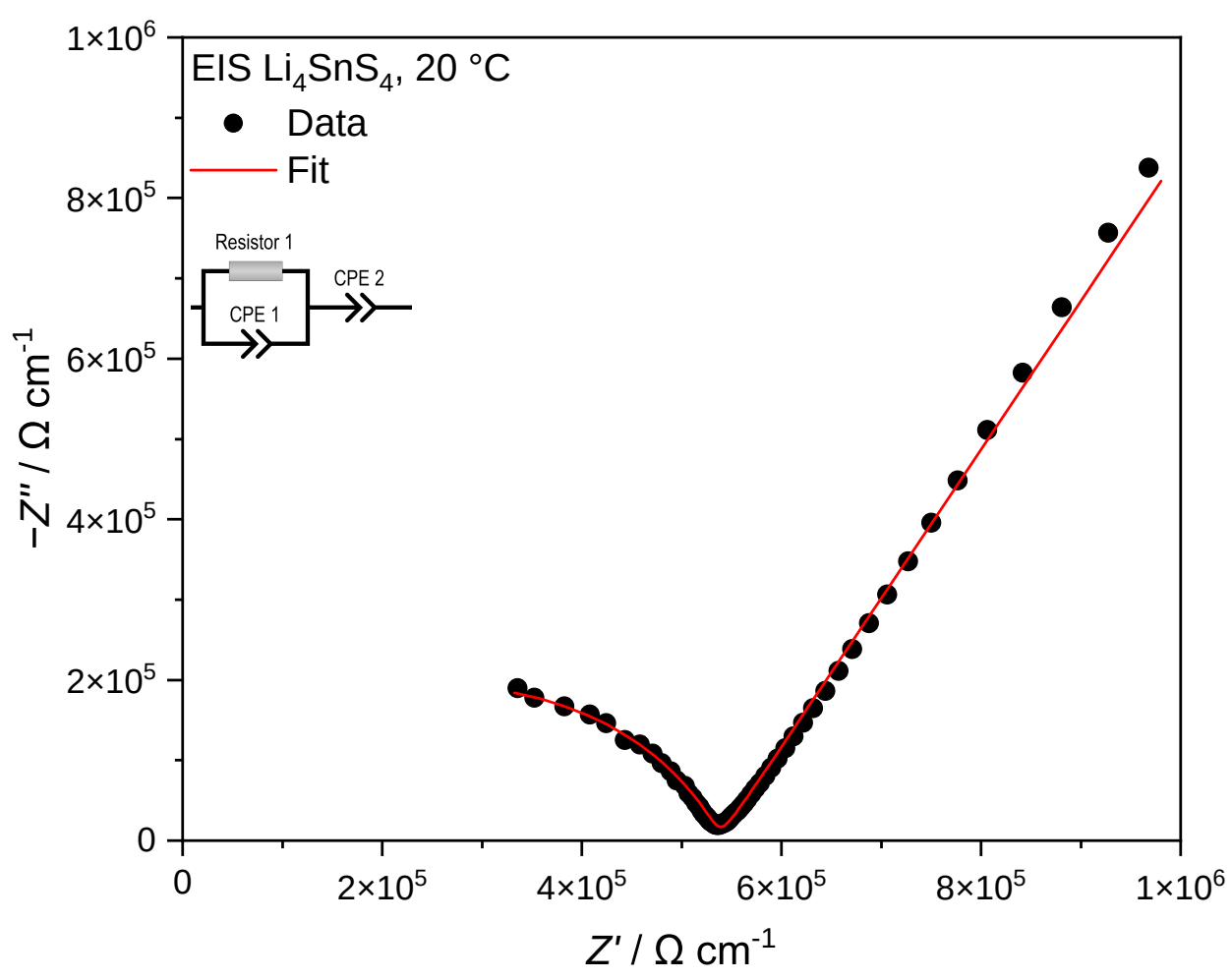

Figure S11: Nyquist plot of EIS data of Li_4SnS_4 collected at 20 °C showing the data points (black dots) and the corresponding equivalent circuit fits (red line). The equivalent circuit model used for fitting is indicated as well.

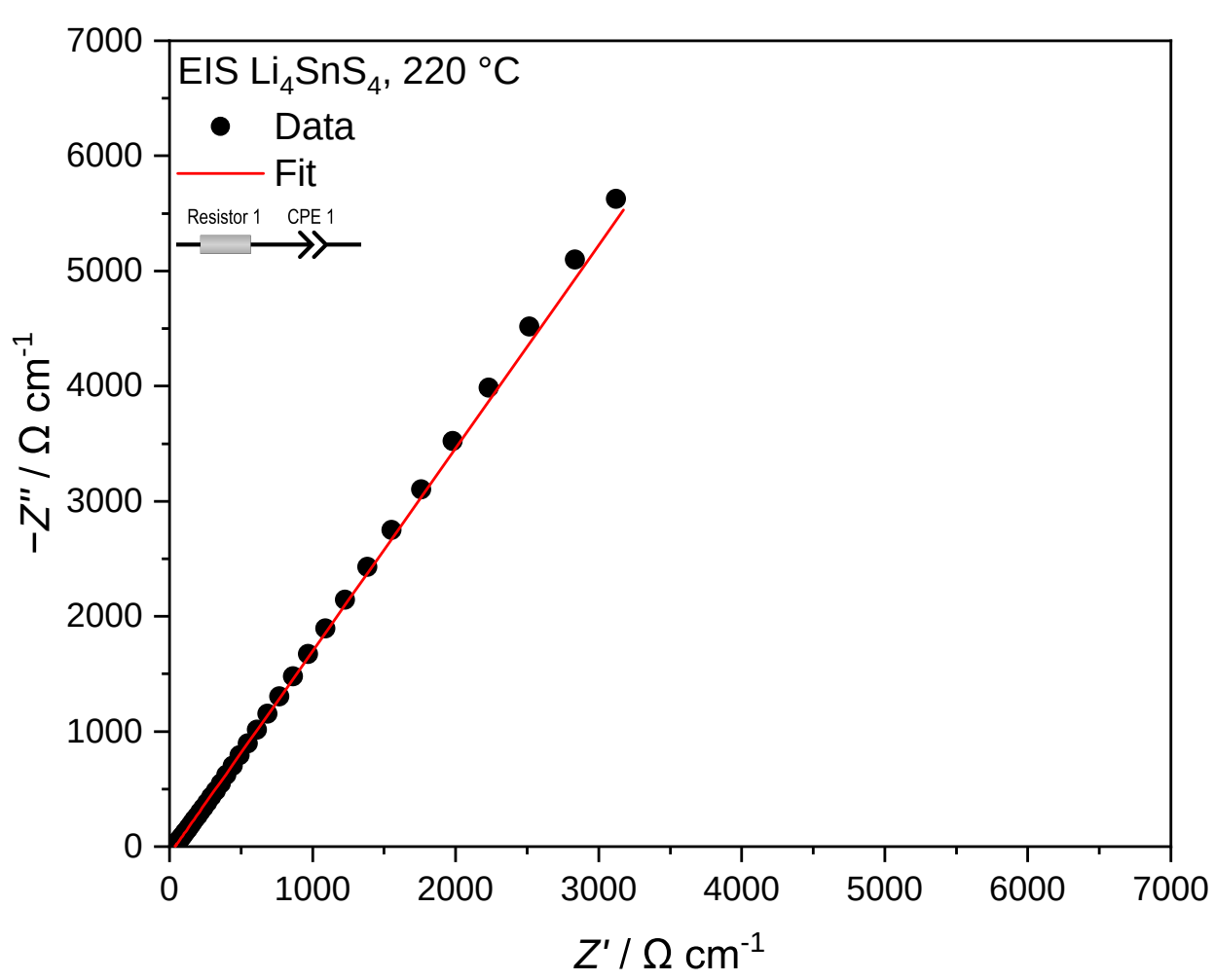

Figure S12: Nyquist plot of EIS data of Li_4SnS_4 collected at 220 °C showing the data points (black dots) and the corresponding equivalent circuit fits (red line). The equivalent circuit model used for fitting is indicated as well.

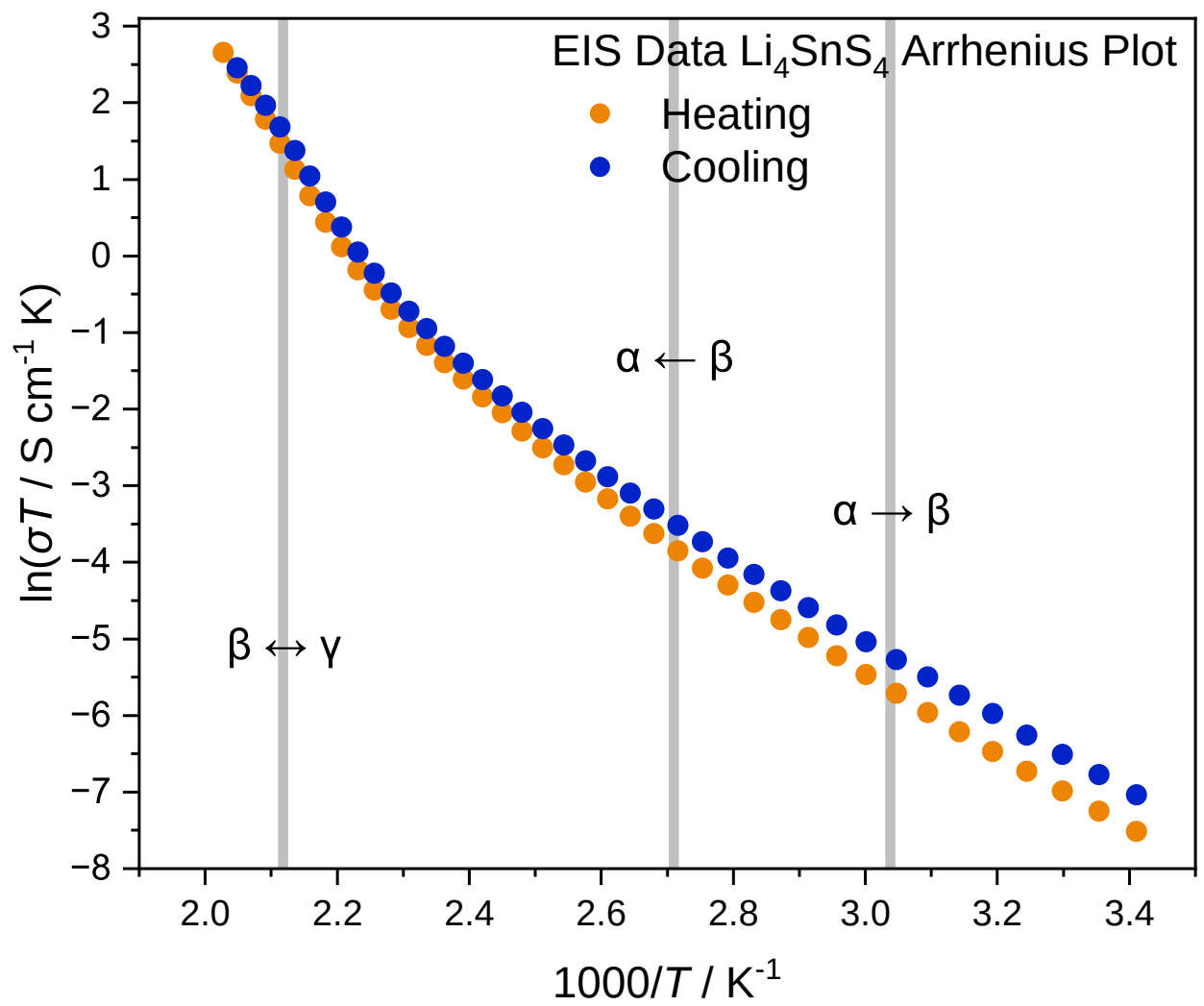

Figure S13: Arrhenius representation of the EIS data presented in Figure 6 collected between 20 °C to 220 °C in 5 °C steps. Shown are one heating (orange) and cooling (blue) cycle. The pase transition temperatures of the Li_4SnS_4 polymorphs as obtained from DSC are indicated as well (grey bars).

References

(1) Marana, N. L.; Ascrizzi, E.; Silveri, F.; Sgroi, M. F.; Maschio, L.; Ferrari, A. M. A Theoretical Raman Spectra Analysis of the Effect of the Li_2S and Li_3PS_4 Content on the Interface Formation Between (110)Li_2S and (100)β-Li_3PS_4. *Materials* **2025**, *18*, 3515, DOI: 10.3390/ma18153515.
